# Meeting report of the OECD conference on “Genome Editing: Applications in Agriculture—Implications for Health, Environment and Regulation”

**DOI:** 10.1007/s11248-019-00154-1

**Published:** 2019-07-15

**Authors:** Steffi Friedrichs, Yoko Takasu, Peter Kearns, Bertrand Dagallier, Ryudai Oshima, Janet Schofield, Catherine Moreddu

**Affiliations:** 1 AcumenIST, Rue Fétis 19, 1040 Etterbeek, Belgium; 2 Organisation for Economic Co-operation and Development (OECD), 2, Rue André Pascal, 75775 Paris Cedex 16, France

**Keywords:** Genome editing, Trade and agriculture, Biotechnology, Safety assessment, Technology regulation

## Abstract

The “OECD Conference on Genome Editing: Applications in Agriculture—Implications for Health, Environment and Regulation” was held on the 28–29 June 2018 at the OECD headquarter and conference centre in Paris, France. It brought together policy makers, academia, innovators and other stakeholders involved in the topic, in order to take stock of the current technical developments and implementations of genome editing, as well as their applications in various areas of agriculture and the implications they give rise to (More information on the “OECD Conference on Genome Editing: Applications in Agriculture—Implications for Health, Environment and Regulation” can be found on the OECD Genome Editing hub: http://www.oecd.org/environment/genome-editing-agriculture/; the hub also contains the detailed conference programme, the biographies of all conference speakers, the detailed conference abstracts, and the presentations of the two-day conference). The conference aimed to provide a clearer understanding of the regulatory considerations raised by products of genome editing, pointing towards a coherent policy approach to facilitate innovations involving genome editing.

## Background

Genome editing refers to techniques, in which specialised enzymes that have been modified, can insert, replace, or remove DNA from a genome with a high degree of specificity. Genome editing, and one of its most discussed techniques, the CRISPR/Cas9 system, has received increasing attention in the academic press and the wider media. This advanced form of genetic engineering provides tools at relatively low cost for innovation in biomedicine, agriculture, industrial biotechnology and other sectors relating to the bioeconomy.

Genome editing has already been successfully used with agricultural organisms of commercial importance, such as agricultural crops and farm animal husbandry, improving the efficiency of plant and animal breeding, and offering the possibility of new methods for the control of pests and diseases. The rapidly growing use of genome editing has policy implications and instigates human health and environmental safety considerations.

## Genome editing in the context of OECD work

The conference formed the pivotal element of the OECD project on “Health and Environmental Safety in Genome Editing Applications” that had previously been initiated by the OECD Council (the OECD’s governing body, made up of the permanent representative of each OECD member country plus that of the European Commission). Upon OECD Council mandate, the project was implemented with support of the OECD’s Central Priority Fund (CPF), which is reserved for addressing topics that have multidisciplinary components and that would thus not otherwise be covered in one particular OECD committee’s work programme. In choosing this special format to conduct a timely project on the topic of one of today’s fastest developing technological innovations, the OECD Council had recognised the immense impact that genome editing is forecast to have in a wide range of research and economic areas. Within this context, the conferences aimed to contribute to OECD work on several issues: science and technology policies including the innovation strategy; food and agricultural policies; environment, health and safety.

The OECD CPF project on “Health and Environmental Safety in Genome Editing Applications” represents a joint effort of the OECD Directorates of Environment (ENV), Science, Technology and Innovation (STI), Trade and Agriculture (TAD) and Public Governance and Territorial Development (GOV), with specific support from the OECD’s Internal Co-ordination Group for Biotechnology (ICGB), which is coordinated by the Environment Directorate. In addition, the “OECD Conference on Genome Editing: Applications in Agriculture—Implications for Health, Environment and Regulation” was supported by the OECD’s Co-operative Research Programme (CRP).

The conference decidedly focussed on applications of genome editing in the agricultural sector, including any associated health and environmental safety considerations; within the context of the conference, the term “health” referred to food and feed safety.

The conference received special guidance and input from a steering group that had been set up with the aim of developing a balanced conference agenda. The steering group comprised 24 participants nominated by OECD delegations to one or more of the following OECD bodies: Working Party on Biotechnology, Nanotechnology and Converging Technologies (STI); Working Group on the Harmonisation of Regulatory Oversight in Biotechnology (ENV); Working Group for the Safety of Novel Foods and Feeds (ENV); and Co-operative Research Programme (TAD).

## The conference agenda

The conference commenced with a wide-sweeping introduction to the current global applications of genome editing techniques (including medical applications), in order to provide a scientific grounding to the subsequent 2 days of detailed discussion on the use of genome editing in agriculture and the potential safety and regulatory issues arising from it.^1^

Following a description of the role and work of the OECD on the topic of genome editing, and a detailed introduction to the global developments of genome editing in agriculture, the conference discussions were organised into three main sessions:

- *Session 1: Applications of genome editing in agriculture*— *plant and animal breeding* This session aimed to take stock of the numerous applications and the wide variety of genome editing technologies that were currently underway; the contributions were provided by expert scientists in the relevant fields of application.
- *Session 2: Risk and safety considerations* This session was designed to highlight potential risks arising in the context of environmental health and safety, and to commence a discussion on the question, if the appropriate tools for the risk assessment of genome editing in agriculture were available.
- *Session 3: Regulatory considerations* This session invited government representatives from countries around the world to report on the regulatory status of agricultural biotechnology in their jurisdictions.

Each session started with a set of presentations by invited experts, who were asked to address specific questions and topics in their contributions, in order to set the scene for an extended round of panel discussions at the end of the session.

## Conference welcome

The conference was opened by Masamichi Kono, Deputy Secretary-General of the OECD. He welcomed the over 200 participants from 35 countries that were representative for the timeliness of a discussion on the multidisciplinary and global issue of genome editing, brought together by the convening, diplomatic power of the OECD. He pointed out the immense innovative power that genome editing presented (and in some cases already provided) to a wide range of economic sectors, and highlighted the significant effect of the technology, especially in the context of its application to trade commodities.

Kono alerted the conference participants to their joint responsibility in addressing the central question of the conference over its 2-day duration:

Should genome-edited organisms be regulated like other genetically modified or engineered organisms, or should different approaches be used?

(Masamichi Kono, “OECD Conference on Genome Editing: Applications in Agriculture—Implications for Health, Environment and Regulation”, 28–29 June 2018)

Ken Ash, Director of the OECD Directorate on Trade and Agriculture, remarked on the unique ambition of the OECD’s Co-operative Research Programme (CRP) to “introduce policy guys to science and science guys to policy”, and welcomed the conference participants, whose diverse background reflected a fulfilment of the CRP’s ambition. He informed the conference delegation how, just 2 years earlier, a congregation of agricultural ministers, representing 48 countries and the combined power of 80% of global agricultural output and trade, had agreed that the current agricultural policies were “not particularly fit for purpose” regarding the need to drive sustainability and productivity and to offer long-term opportunities to the global agricultural industries (OECD 2016a, b).

Ash reminded the conference of the interdisciplinary nature of both science and policy and outlined his expectation of this and other discussions on genome editing to recognise and address the concerns that people might harbour regarding this new technology, because the mitigation of differences up front was easier than the undoing of previous decisions. He ultimately tasked the conference with addressing the challenge to “find a way to move forward—together rather than separate—and allow science to contribute to what will otherwise be an impossible ambition: to feed a growing world with […] a share of an awful lot less resources than we enjoyed in the past!”

## Introductions to the field of genome editing

Fyodor Urnov, Deputy Director of the Altius Institute for Biomedical Sciences, the United States, provided an overview of what he called “the global footprints of genome editing through the particular prism of therapeutics”, predicting that the present audience of agricultural experts would recognise a large overlap of their issues of genome editing with those raised in the field of human medicine. Urnov noted that the technique of genome editing was no longer an aspirational goal, as genome editing had already been successfully applied to numerous plant and animal species, as well as to human patients.

He introduced the discovery and development of genome editing techniques by illustrating a timeline of the technology’s advancement during a period that he called “B.C. (Before CRISPR)”, and which designated the technology’s development until 2012: the original proof of concept that a genome could be edited with the help of programmable nucleases had been established in the early 2000s with work on model organisms, such as using frog oocytes and flies (Bibikova et al. 2002). At that time, so-called zinc finger nuclease (ZFN) had been the editor of choice. In 2005, ZFN genome editing had been applied to human cells and named “genome editing” by a team of researchers at Sangamo Biosciences (USA) (Urnov et al. 2005), followed by its first deployment in an aquatic species (zebrafish) in 2008 (Doyon et al. 2008), in plants (maize) by Dow AgroSciences in 2009 (Shukla et al. 2009), in rats (Geurts et al. 2009), pigs (Hauschild et al. 2011), human embryonic stem cells (Lombardo et al. 2007), and rabbits (Flisikowska et al. 2011).

In 2011, a new genome editor had entered the scene: the transcription activator-like effector nuclease (TALEN) promised a higher specificity than the ZFN-based approach, but widespread application had continued to be hampered by the difficulty in synthesising the zinc-finger and TALE nucleases that had required immense effort. A significant breakthrough in the quest of finding a more easily accessible and deployable editor had finally been achieved in 2012, when it was discovered that the nuclease Cas9, guided by RNA (ribonucleic acid), allowed everything that had been known about ZFN and TALEN to also be deployed for CRISPR (clustered regularly interspaced short palindromic repeats)/Cas9 systems.

Urnov explained that all genome editing techniques relied on the single step of engineering an enzyme (i.e. the nuclease), which induced a double-strand break (DSB) at a specific site of the DNA that was to be edited. He highlighted that the beauty of the suite of genome editing techniques lay in the fact that it did not matter, which of the different known nucleases (ZFN, TALEN, meganucleases or megaTALs, or CRISPR RNA-guided nucleases) was used to induce the DSB, because the biological outcomes would always be the same. Nevertheless, the CRISPR/Cas system, which was guided by support of a piece of RNA, had become the most widely used system, because the guide RNA could be designed by “basic high-school biology”.

Urnov went on to explain the principles of the genome editing process by pointing out that a DSB was a highly genotoxic lesion, for which Mother Nature reserved two different ways of repair: the simplest way was to put the ends of the DSB back together again through non-homologous end-joining (NHEJ), but repair errors could happen with a short fragment of DNA residues removed from or added to the site of the break, resulting in the disruption of the gene function. These types of genetic alterations were also observed in natural mutations. The second way of DSB repair was called “homology-directed repair (HDR)”, which used a sequence donor, part of which was homologous to the DSB site. He continued to describe how scientists harnessed this naturally occurring repair mechanisms by providing a repair template. He repeatedly emphasised that no memory of gene editors or repair templates remained in the cell, and that the DNAs provided to the cell were not transferred to the chromosome, while just information was transferred to the chromosome.

Urnov summarised his explanation by illustrating the main applications of the genome editing toolbox (Urnov et al. 2010):

1. *gene knockout*: gene disruption by introducing small deletions or insertions (by NHEJ);
2. *tagging genes*: targeted introduction of a nucleotide adaptor with sticky ends complementary to the DSB ends through tag ligation by NHEJ;
3. *study of larger genomic regions*: targeted introduction of a large deletion (of over 100 bp) by NHEJ;
4. *gene correction*: introduction or repair of point mutations by HDR;
5. *targeted gene addition*: introduction of transgenes by HDR; and
6. *transgene stacking*: targeted introduction of multiple transgenes by HDR.

In a brief aside on the question of intellectual property (IP) protection concerning genome editing, Urnov noted the overwhelming commercial interest in the novel genome modification technique: he explained that the ZFN technology was the exclusive property of Sangamo Biosciences, and that it had been licensed to the Sigma-Aldrich and Dow AgroSciences collaboration for research purposes. The TALEN technology was owned by Calyxt for application in plants, by Cellectis for therapeutics, by Recombinetics for animals, and by Thermo for research applications, while the ongoing patent dispute about the CRISPR/Cas9 technology left all assignment of technology ownership open for (probably) several years to come. Urnov noted that an important point on genome editing from an intellectual property perspective was that the know-how of making the genome editor was essentially in the public domain:

There are no trade secrets at this point in this field; a high-school student should be able to put together a genome editor using the CRISPR/Cas9 system.

(Fyodor Urnov, “OECD Conference on Genome Editing: Applications in Agriculture—Implications for Health, Environment and Regulation”, 28–29 June 2018)

Urnov called upon the scientific community not to worry about IP on the technology itself; in his regard, IP was “not a legitimate obstacle on the path of a good global footprint of genome editing; […] it is too versatile, too broad and too many people are working on it.”

Urnov concluded the technical part of his presentation by re-emphasising how genome editing had redefined the meaning of the word “natural”, because it allowed the transfer of a natural form of a gene from one living organism to another without adding an extra gene to the recipient; during this process, the recipient’s own natural gene was changed to a different equally natural form. Stressing the unprecedented ability of genome editing to cleanly introgress haplotypes (without any back-crossing or without the need to clean up genetic background), Urnov described a number of clinical research trials that used genome editing, and provided his own predictions on the possible paths forward for each one of the trials; he forecast the following step-wise advancement paths of clinical genome editing, which would ultimately lead to a versatile, global footprint of the technology:

1. HIV:
  in 2–3 years: continued efforts on ex vivo edited autologous T-cells and hematopoietic stem and progenitor cells (HSPCs) would achieve a demonstration of good safety records;
  in 5 years: the first approved genome editing medicine (i.e. autologous T-cells or HSPCs for HIV) would be available;
  in 5–7 years: ex vivo genome editing vaccines (i.e. autologous) would be readily available; and
  in 10 years: in vivo genome editing vaccinations would be readily available.
2. Cancer:
  in 1–3 years: clinical trials with edited cells for leukaemia would take place;
  in 1–5 years: clinical trials with edited cells for solid cancers would take place;
  in 2–5 years: clinical trials with edited off-the-shelf (i.e. cells donated by healthy donors) cells would take place; and
  after 5 years: off-the-shelf edited cell medicines against cancer should be much cheaper than the costs of current one-off clinical trials.^2^
3. Haemoglobinopathies:
  in 1–5 years: clinical gene therapy trials would be continued to broaden the scope of the currently conducted conventional gene therapies through the application of genome editing technologies (i.e. the first approval could be expected in 2019, but it would be an expensive procedure);
  in 1–6 years: clinical genome editing trials would continue;
  in 5–7 years: the first approval of a genome-edited medicine could be expected; and
  long-term goal: if a way was developed to deliver the genome editor to stem cells by in vivo injection, a scale-up to Asia and Africa would be possible.

To illustrate the rapid advancement that was already taking place in the field of clinical genome editing, Urnov illustrated the case of baby girl Layla: in November 2015, scientists in the UK applied the genome editing technique to save the girl that had been dying from leukaemia, by giving her off-the-shelf allogeneic T-cells that had previously been donated by a healthy volunteer and then engineered by the genome editing team to have specific properties. He concluded that the use of allogeneic cells was the way forward to a wide-spread, affordable deployment of genome editing vaccines.

Urnov summarised by listing those diseases, in which clinical genome editing could already make significant contributions to public health; these included numerous forms of cancer, HIV, haemoglobinopathies and metabolic diseases. He predicted that the most widespread global footprint of clinical genome editing would probably be delivered as vaccines: “I […] think that a fundamental global impact of this technology will lie—ultimately—not only in the treatment of disease but in its prevention by pre-emptive genome editing. And that, of course, leads to the very thorny issue of genome editing for human enhancements,” he noted.

Urnov finished his presentation by alerting the audience that one of the largest issues human medical genome editing had to grapple with was that of human enhancement: he provided examples of proven genetic mutations that allowed significant enhancements of certain properties to those affected by them: a known genetic mutation that enabled humans to require just four hours of sleep per night could be an ideal enhancement for air traffic controllers (He et al. 2009), while a natural mutation that led to the loss of pain sensation could be deployed for pain-free special-force soldiers (Drenth and Waxman 2007).

Genome editing allows you to move a natural form of a gene from one living organism to another without adding an extra gene to the recipient; so, you solely change the recipient’s own natural gene to a different equally natural form.

(Fyodor Urnov, “OECD Conference on Genome Editing: Applications in Agriculture—Implications for Health, Environment and Regulation”, 28–29 June 2018)

He concluded that there was no conceptual obstacle to first trying something for disease treatment and transitioning it to disease prevention. “This is technically feasible, the question is: is this legitimate ethically?” he added.

## The OECD’s role and activities in genome editing

Peter Kearns of the OECD provided an overview of the international organisation’s activities related to modern techniques of biotechnology and genome editing: two active programmes were currently addressing the OECD’s central aim regarding genetically engineered organisms (GEOs) or genetically modified organisms (GMOs) (Kearns 2019):

Aim: Help to address human health and environmental safety issues, through science-based risk assessment, for products of modern biotechnology (GEOs): plants, animals, micro-organisms.

(Peter Kearns, “OECD Conference on Genome Editing: Applications in Agriculture—Implications for Health, Environment and Regulation”, 28–29 June 2018)

Kearns outlined the OECD work programmes and the central questions they were addressing:

1. *environmental safety of GEOs (biosafety)* through the “Working Group for the Harmonisation of Regulatory Oversight in Biotechnology”:
  Central question: Which features should environmental risk assessment be based on?
2. *foods/feeds derived from GEOs* through the “Working Group for the Safety of Novel Foods and Feeds”:
  Central question: Is this new food/feed *as safe as* its conventional counterpart?

Both groups were composed of bio/food safety National Authorities (i.e. regulators, risk assessors experts), as well as observers from other organisations.

Kearns described the basic principles of both the purpose and the means of the OECD’s biosafety work:

*Purpose of the OECD’s biosafety work:*

- assist countries to evaluate potential risks of modern biotechnology products for human/animal health and environment, and ensure high standards of safety;
- limit duplicative efforts: mutual understanding, acceptable data; and
- reduce the potential for non-tariff barriers to trade.

*Means of the OECD’s biosafety work:*

- harmonisation of approaches and regulatory frameworks; and
- share/disseminate common base of scientific information.

He explained that the main outputs of the OECD’s work on biosafety could take the form of: (a) exchange or cooperation meetings, conferences or workshops, during which biosafety authorities of the OECD member countries and its guest members discussed current and upcoming issues, (b) “consensus” and guidance documents that helped national assessment and decision-making processes, and that represented practical tools for comparing conventional and “genetically engineered” products,^3^ and (c) a database on transgenic products that had been approved for release in the environment and/or food and feed use.^4^

Kearns also alerted the audience to a previous workshop, organised by the OECD Working Party on Biotechnology, Nanotechnology and Converging Technologies (BNCT), held on the 29–30 September 2016 in Ottawa, Canada. Under the title “Gene Editing in an International Context: Scientific, Economic and Social Issues across Sectors”, the workshop had aimed to encourage an information exchange and facilitate a cross-disciplinary discussion on the science, governance, and economics of genome editing innovations in all application areas (i.e. applications in agriculture and aquaculture, environmental applications, and applications in human medicine). The full workshop report had been published in March 2018 (Shukla-Jones et al. 2018).

## Global developments of genome editing in agriculture

Agnès Ricroch, Professor in Evolutionary Genetics and Plant Breeding at the AgroParisTech, France, and Adjunct Professor at Pennsylvania State University, the United States, provided an overview of the global developments of genome editing in agriculture (Ricroch 2019). She commenced her presentation by outlining the current most pressing ethical considerations in agriculture:

- global food production needed to increase as much as 70 percent to support the growing population in the world (10 billion in 2050);
- it was important to consider whether and how genome editing technologies could contribute to improving the efficiency of food/feed distribution and reducing waste;
- the safety of food for human consumption was a key concern; and
- in the case of animals, there were also concerns about the welfare of farmed animals.

Ricroch then set out to discuss the potential impacts that the variety of different genome editing tools could have on the highlighted ethical considerations in agriculture. In particular, she raised the questions concerning the potential impact of applying genome editing techniques in agriculture:

- Could genome editing lower the cost of animal and plant breeding methodologies? Could it make breeding processes faster?
- Would genome editing raise new environmental health and safety challenges?
- Could genome editing have an impact on sustainable development?
- What could the consequence of the technology’s application be for farmers?
- Could its application have an impact on consumers, society and/or the environment?

She referred to the history of the developments in genome editing that had been presented by Fyodor Urnov, and specifically emphasised that the discussion about the potential impact of genome editing technologies (on agriculture) had recently become a timely topic, due to the step-change that the discovery of TALEN and CRISPR/Cas9 techniques had brought about: while the first genome editing approaches were long, expensive processes, the new ones represented a group of relatively low-cost tools that could be designed in weeks (in the case of TALEN) or days (in the case of CRISPR) (see Table 1).

**Table 1:** Properties of genome editing tools (after Agnès Ricroch)

The four families of gene editors (first year of report)	Meganucleases (1985)	Zinc finger nucleases (2003)	TALENs (2010)	CRISPR/Cas (2012)
Number of proteins	1	2	2	1 + 1 RNA
Production	Difficult	Not very easy	Easy	Very easy
Cost of production	50,000 Euro	5000 Euro	1000 Euro	10 Euro
Time needed for an experiment	Months	Months	Weeks	Days

Ricroch summarised that genome editing tools had thus advanced to a democratic method:

The low cost and the fast production allow not only private companies and multinationals to develop new biotech crops and animals, but also public–private groups—especially in developing countries—consortia with non-profit ends.

(Agnès Ricroch, “OECD Conference on Genome Editing: Applications in Agriculture—Implications for Health, Environment and Regulation”, 28–29 June 2018)

She noted that the new genome editing techniques enabled researchers to work with the native DNA and enhance what natural evolution had provided; by using a simple “cut and paste” and “find and replace” approaches, the sites of the genome linked to specific traits could now be precisely edited. Numerous research articles demonstrating the potential use of the CRISPR genome editing technology in agriculture had already been published: the largest number of papers had been published by researchers from the People’s Republic of China (hereafter “China”) (46% of all CRISPR articles with agricultural applications), followed by the United States (25% of all CRISPR publications with agricultural applications) (Ricroch et al. 2017). Not surprisingly, the specific agricultural plant or animal that the research papers focussed on varied heavily between the countries, reflecting the consumption behaviour, as well as the scientific and economic contexts in the relevant country.

Ricroch continued to illustrate a number of predictable benefits that the application of genome editing techniques in agriculture would bring to consumers, (organic) farmers, farm animals, the agricultural industries, and patients:

- *Benefits for farmers*:
  - The genome editing of tolerances to abiotic stress (e.g. drought, cold, high salinity, flooding, extreme temperatures, nitrogen deficiency) could provide *solutions to* the challenges that *climate change* posed to farm crop.
- *Benefits for organic farmers*:
  - Edited crops, thanks to their potential to increase *sustainable farming practices*, could be cultivated by organic farmers and therefore could fall within the ethos of organic agriculture.
- *Benefits for farm animals and farmers*:
  - The genome editing to modify the susceptibility to animal diseases (e.g. African swine fever (ASF), porcine reproductive and respiratory syndrome (PRRS)) could prevent diseases in farm animal populations, and thus *avoid animal suffering* (or even the need for the culling of diseased animals), *medical treatment* of animals (e.g. with antibiotics), and the resulting *vast economic losses* to the farmers.
  - The editing of the hornless gene of Black Angus cattle into that of Holstein–Friesian dairy cows provided an example for the possible *prevention of injuries and suffering of farm animals.*
- *Benefits for consumers*:
  - The prevention of food spoilage using genome editing was demonstrated through examples, such as white button mushrooms, apples or potatoes that are resistant to browning (Waltz 2016). They represented a significant advancement in the *avoidance of food waste*, which—in itself—posed a major challenge to both the developing and the developed world.
  - The genome editing of potatoes could result in a targeted reduction of the formation of acrylamide in the tuber when baked, fried or roasted at high temperatures, thus providing a significant reduction of a natural food chemical that was suspected to have negative health effects (Waltz 2015). Other *increased nutritional traits* that could result from gene editing included the lowering of the gliadin content in wheat, the increase of the fibre content in wheat, the increase of the oleic oil contents in soybean and camelina (Calyxt, TALEN^®^), the increase of the amylose content in rice, and the increase of the anthocyanine production in grapes.
- *Benefits for consumers and farmers*:
  - The genome editing to introduce biotic stress resistance (i.e. a resistance against pests, diseases and weeds) into plants could *prevent vast economic losses* and *food safety threats*.
- *Benefits for consumers and industry*:
  - The first large-scale industrial use of the CRISPR genome editing technology to increase the beneficial starch content in waxy corn from 75 to 100%, as announced by DuPont Pioneer in April 2018, had provided an example of an *improved product* to the consumer, while simultaneously *increasing the yield and productivity* of the industry.
- *Benefits for patients*:
  - The genome editing enabled modification of large animals that served as sources for xenotransplantation organs represented a major advancement in the transfer of tissues and organs from animals to *treat organ losses or dysfunctions in humans* (Reardon 2015).

Ricroch summarised that the main benefit that genome editing promised to all stakeholders was its capacity to significantly speed up the progress of breeding programmes: from 7–25 to as few as 2–3 years. The target-specificity of genome editing techniques could effectively bypass the need to go through a number of plant generations to achieve a particular genetic combination to obtain a desired beneficial trait.

She concluded her presentation by alerting the audience to those parameters that were needed, in order to advance and adopt genome editing in agriculture, and to thus address sustainability for society (Ricroch et al. 2016):

- Biosafety protocols were required, especially for novel technologies. But the protocols should be adapted to each trait, be flexible (dynamically scalable), and revisable.
- Furthermore, *ex ante* risk assessment should, whenever possible, be gradually replaced by the adoption and monitoring of good agricultural practices, as well implementing precision agriculture.
- The implementation of good agricultural practices (GAP) would be crucial; GAP helped improve food, environmental and occupational health and safety, and were a key factor in sustainable agriculture.
- Farmers should be adequately informed and prepared to use edited crops or animals more efficiently and sustainably, which could help reduce the use of pesticides, herbicides, fertilisers or antibiotics.
- Genome editing, particularly the CRISPR system, had spread rapidly through the biological sciences. It could speed up animal and plant breeding.
- Animals with better feed efficiency and welfare, and plants with greater productivity and adaptation climate change helped the global sustainability.

During the question and answer session, it was noted that certain desired traits had been achieved using conventional (transgenic) genetic engineering methodologies, as well as through the application of novel genome engineering techniques: for example, the acrylamide-content in potatoes that were processed at high temperatures could be reduced either by (a) introducing a dsRNA-expressing transgene into the potato genome, or by (b) using the TALEN genome editing technique that did not leave foreign DNA behind. Ricroch noted the method was not relevant to the consumer, who got to benefit from the improved properties: “What is important to the consumer is a safe product, whatever the method that brings this safety and this product to improve our daily lives. The best was to evaluate the risk […], based on the trait [and] on the end product, [rather] than on the method,” she added.

She noted that the new consumers (e.g. the Millennials) were very aware of the impact of agriculture on biodiversity, greenhouse gas emission, so that the genome editing community had a responsibility to tell the consumer that genome editing speeded up the breeding programme, not consuming so much energy.

The delegation concluded, however, that the advent of novel genome editing techniques had not rendered conventional (transgenic) genetic engineering technologies obsolete: the application of genome editing was limited, because it necessitated gene sequencing of the organism that was to be modified; genome editing could not be applied to plants and animals whose genomes had not been sequenced. In addition, some desired traits required changes in large DNA sections that could best be achieved through the inclusion of specific and ready characterised sequences; in these cases, conventional transgenic approaches were still the method of choice.

We should think about a wide range of tools in the [genetic engineering] toolbox to take the best tool to make the trait that farmers, consumers and industry need to improve the lives of people.

(Agnès Ricroch, “OECD Conference on Genome Editing: Applications in Agriculture—Implications for Health, Environment and Regulation”, 28–29 June 2018)

## Session 1: Applications of genome editing in agriculture

This session highlighted case studies and examples of agricultural applications of genome editing, particularly plant varieties and animal breeds that may be on or close to the market or under research and development.

The session was introduced and moderated by Elselien Breman from the European Agricultural and Fisheries Policy and Food Security Department at the Ministry of Agriculture, Nature and Food Quality in the Netherlands.

### Agricultural applications of genome editing: crop plants

Caixia Gao from the State Key Laboratory of Plant Cell and Chromosome Engineering, Institute of Genetics and Developmental Biology, Chinese Academy of Sciences (China) introduced the topic of “Precision plant breeding using genome editing technologies” (Gao 2019). She outlined the advantages and novel opportunities that genome editing techniques had brought to the field of crop breeding by comparing the different approaches known to the field; genome editing was a rapid and precise breeding method that provided predictable variations, while the other breeding techniques needed higher cost and/or longer time to obtain final products.

Gao noted that DNA-free genome editing using ribonucleoprotein complexes (RNPs) or in vitro transcripts (IVTs) overcame several significant disadvantages of conventional DNA-based genome editing techniques in crop breeding, including: (a) the potential off-target effects, (b) the time-consuming segregation steps, and (c) the problem to insert small DNA fragments. She emphasised however, that the most important and unique drawback of DNA-based genome editing compared to DNA-free genome editing was that the former was impossible to segregate away the transgene in vegetatively propagated plants, such as bananas (Zhang et al. 2016).

Gao provided several examples of the use of genome editing techniques; these included the following:

- the introduction of resistance to powdery mildew in the hexaploid wheat, which was otherwise known to be recalcitrant to conventional breeding techniques (Wang et al. 2014);
- the acceleration of the breeding of corn (Li et al. 2017); and
- the editing of the flavour of rice (Shan et al. 2015).

By illustrating a recent example of a single-base substitution, Gao pointed out the high-precision capability of the base editing technique (Komor et al. 2016); in this example, the researchers had used the base editor, a cytidine deaminase fused to dCas9, in order to mediate a conversion from cytidine to uridine, introducing herbicide resistance in rice.

Gao summarised her presentation by drawing the following conclusions:

- Genome editing could effectively induce targeted mutations in plant genomes:
  - at a precise location;
  - with many alleles at the same time, also in polyploid crops;
  - with reduced non-specific off-target cleavages;
  - allowing highly efficient and site-specific C-to-T base editing;
  - without foreign DNA remaining in the genome; and
  - allowing to generate identical mutations to the products obtained by ‘conventional’ mutagenesis.

She made the following demands concerning the future economic, regulatory and societal perspectives of genome editing techniques:

- Costs for precise and efficient molecular breeding should be reduced.
- Regulatory requirements should be eliminated or significantly reduced:
  - regulation of products of new breeding techniques (NBTs) should be consistent with products from conventional breeding, if they were indistinguishable; and
  - regulation for safe use should focus on characteristics, phenotype and intended use of the plant.
- Public concerns about gene-edited crops should be alleviated.

Gao highlighted that the attitude of the Chinese public was influenced by a widespread use of technologies in general; she alerted the genome editing community to focus on the benefits of the technique in their communications.

When we talk about genome editing, people should not be thinking about GMO, which is negatively perceived.

(Caixia Gao, “OECD Conference on Genome Editing: Applications in Agriculture—Implications for Health, Environment and Regulation”, 28–29. June 2018)

### Agricultural applications of genome editing: farmed animals

Simon Lillico, from the Roslin Institute and Royal (Dick) School of Veterinary Studies, and the University of Edinburgh (both in the United Kingdom) opened his presentation on “Agricultural applications of genome editing: Farmed animals” by reminding the audience that the modification of livestock and crops was neither a recent development, nor was it dependent on new technologies (Lillico 2019): humans had modified their environment (e.g. developed settlements, cultivated land, mined materials from the ground), their companions (e.g. bred Chihuahuas from wolves), and their livestock (i.e. selected those animals for breeding that fitted best into their agricultural systems) throughout history.

**Fig. 1:**
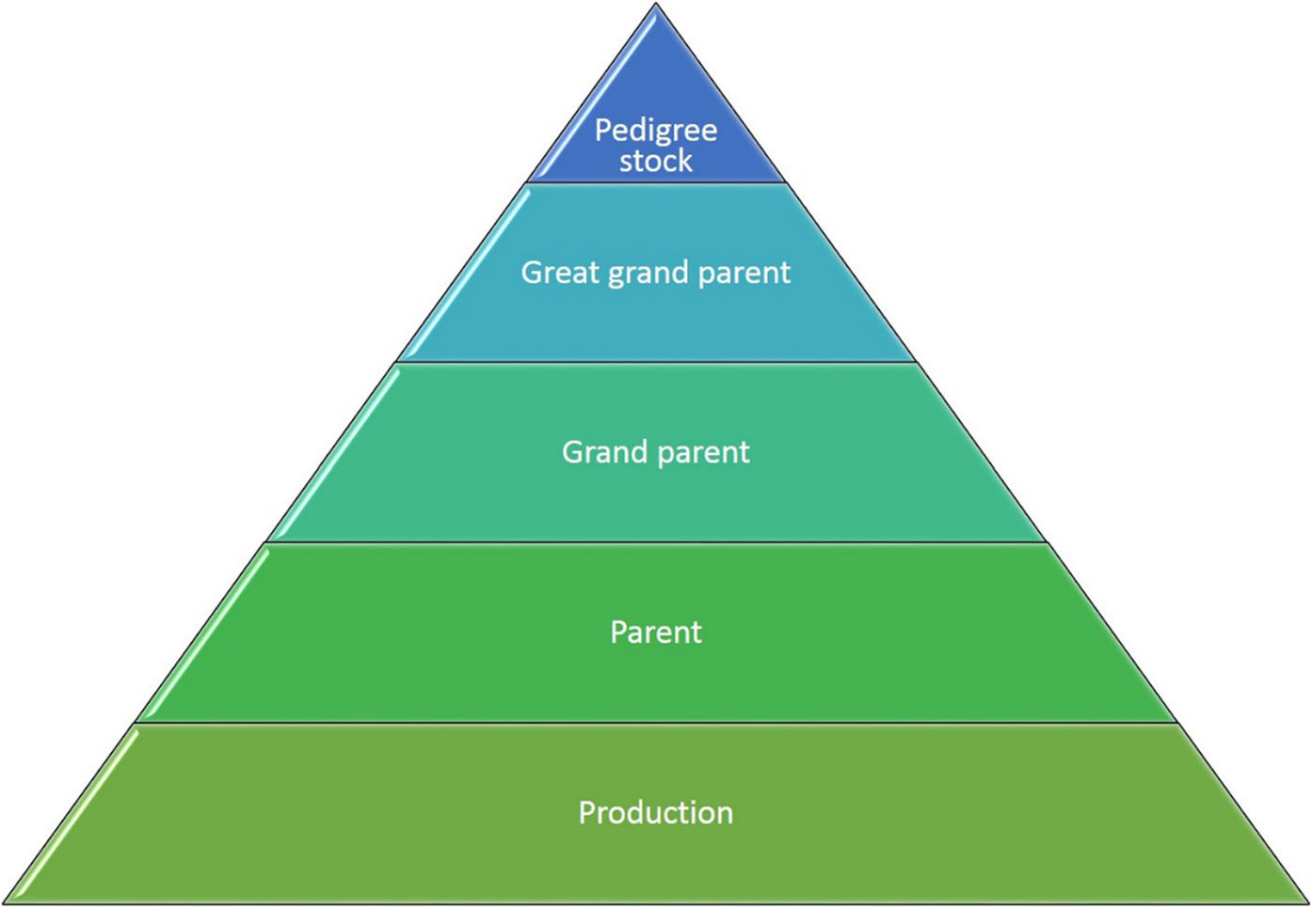
Breeding pyramid (after Lillico, “Agricultural applications of genome editing in farmed animals”, 28 June 2018)

He illustrated the enormous time and resources that were required for the traditional breeding of plants and animals by means of a schematic breeding pyramid (see Fig. 1): in the case of cattle, which represented one of the most time-consuming animals to breed, at least 8–10 years of breeding were required between the selection of the pedigree stock and the commercialisation of the desired product, with typical 2-year generation intervals. In addition, conventional breeding was restricted to existing traits, and made it difficult to introgress new traits into elite stock.

Lillico pointed out that genome editing provided a solution to the challenges of breeding times: genome editing did not need backcrossing procedures that were necessary for conventional breeding to remove many undesirable traits linked to desirable traits, and it also allowed the introduction of one or multiple edits to a pedigree stock in a single generation using cell-based cloning, since many traits were likely to be polygenic. He added another characteristics of genome editing breeding that enable the introduction of traits from related and unrelated species, and even to design entirely new traits, as long as the genome-phenome interaction was sufficiently understood.

He noted that while the tools used for genome editing in livestock animals were identical to those used in humans, the two approaches were separated by severe differences in their intended goals: “In humans, there is an international moratorium on editing the germline. In livestock, we absolutely want to modify the germline.” He added:

We want our [engineered] traits to be heritable generation after generation after generation ….

(Simon Lillico, “OECD Conference on Genome Editing: Applications in Agriculture—Implications for Health, Environment and Regulation”, 28–29 June 2018)

He provided the technical information that supported unprecedented genome editing innovations in the field of animal breeding:

- Chickens had been notoriously recalcitrant to biotechnologists’ abilities to deliver transgene or to modify the genome; germline modification was now possible, however, by the injection of modified primordial germ cells (PGCs) into embryos. The modification of PGCs was also achieved by directly injecting PGCs with gene engineering tools and if necessary, transgenes. Germline chimeras were obtained in the injected generation (G0), and heterozygous offspring were selected in the next generation (G1) to obtain homozygotes in the subsequent generation (G2). The in vitro gene modification of PGCs was technically demanding and resource intensive, while it had an advantage of allowing in vitro selection.
- In fish, germline genome editing was possible by injecting genome editing tools to the single cell that emerged at the top of the newly fertilised egg.
- In mammals, spermatogonial stem cells could be isolated from the testis and cultured in vitro, in order to be genetically modified either with transgenes or with genome editing techniques; the testis was subsequently repopulated with the modified cells to produce genetically modified sperm.
- Gene modification in mammalian zygotes was also achieved by the somatic cell nuclear transfer using in vitro modified fibroblast cells, or by the direct injection of the genome editing reagent into the zygote. In the case of pigs, sheep and goats, the zygotes would subsequently be replanted into the animal for growth and birth. In cattle, by contrast, the fertilisation would be conducted via non-surgical delivery (i.e. with an artificial insemination gun).

Lillico noted that most genetically engineered changes in livestock concerned the improvement of the relevant production trait:

- In general, production traits could be monogenic or polygenic.
- Environmental adaptations were essentially production traits.
- Disease resistance could be regarded as an environmental adaptation, and was essentially an animal welfare issue with immense impact on production.

He described the recent applications of genome editing techniques to introduce disease resistance into domestic pigs:

1. In order to combat African swine fever, which killed 90–100% of infected domestic pigs, scientists had moved a genetic variation across species barriers from the warthog, which was immune to the fever, to the domestic pig; the results of this experiment were not available until the challenge experiment had been done.
2. The porcine reproduction and respiratory syndrome (PRRS), which was carried by a highly mutagenic RNA virus, causing vaccination attempts to typically be slow and ineffective, caused a worldwide economic loss of EURO 1.5 billion to the pig farming industry. Studies had shown that protection against the disease could be achieved by genetically engineering a protein on the surface of the monocytes and macrophages that the virus exclusively bonded to, so that the resulting homozygous pigs produced a modified protein with the binding domain removed, and showed PRRS resistance. Without genome editing techniques, the required alteration was not possible, because the protein itself was very important for haemoglobin/haptoglobin recycling processes; if the entire gene was knocked out, or rendered dysfunctional, haemoglobin would gather in the tissue of the animal.

Lillico mentioned anticipated developments of the CRISPR technology in animal breeding: the use of Cas-like enzymes such as base editors without incorporating double stranded breaks, and RNA editing to specify beneficial genes and modifications. He concluded by alerting the audience that the application of genome editing required adequate care and precautions:

[With] any modification we make, there is the potential of an unintended one.

(Simon Lillico, “OECD Conference on Genome Editing: Applications in Agriculture—Implications for Health, Environment and Regulation”, 28–29 June 2018)

### Examples of specific genome editing products in agriculture

During the last part of “Session 1: Applications of genome editing in agriculture”, eight presentations described specific products under development, demonstrating the types of modifications introduced into plants and animals through genome editing techniques, and how such modifications had been achieved. The presenters described the process from discovery to final product, including proof of concept, as well as the early and advanced development processes. Some presentations covered the breeding steps taken between the initial editing event and the final product, as well as the characteristics and performance of the product.

### DNA-free genome editing with CRISPR enzymes in crop plants

Sunghwa Choe from the School of Biological Sciences at Seoul National University, Korea described the process of “DNA-free genome editing with CRISPR enzymes” (Park and Choe 2019). He illustrated that the reason for his research on this topic had been the tedious and insufficient nature of the traditional approach of gene discovery by T-DNA (transfer DNA) random mutagenesis. During this approach, that was commonly used to understand the function of a gene, around 100 000 T-DNA-inserted mutants were made, which covered 80% of genes, but only 10–20% of the resulting populations exhibited an effect in the phenotype, because gene functions typically complemented each other in plants. Researchers had thus needed multiplex gene mutations for a long time. Genome editing techniques in general provided a significant advancement to this process, enabling the insertion, deletion and replacement of specific sequences in a plant genome, irrespective of the genome editor used.

Genome editing techniques that utilised endonuclease in the form of DNA, however, would always remain prone to falling under the regulation of transgenic methods, and thus require up to 10 years to receive potential market approval of the resulting “transgenic plant” (Camacho et al. 2014). Choe’s team had thus focussed on the ability to deliver the CRISPR/Cas9 editor into plant cells through non-transgenic means, such as the transfection of ribonucleoprotein (RNP), consisting of Cas9 protein and single guide RNA, into calli or protoplasts.

Choe’s team demonstrated that this method worked in rice, lettuce and* Arabidopsis*. In the latter plant, he had also been able to demonstrate the negligibility of off-target effects, because a subsequent deep-sequencing revealed that both the RNP-based genome editing experiment and the RNP-free control group exhibited the same level of off-target edits.

He further compared four different ways of CRISPR/Cas9 delivery: (a) transgenesis using T-DNA, (b) transient DNA transfection, (c) transient mRNA transfection, and (d) transient RNP transfection. He concluded that RNP transfection had achieved the highest mutation efficiency and the highest specificity of genome editing in a shorter timeline compared to T-DNA-based transgenesis, without foreign gene integration as well as antibiotic selection.

He summarised his presentation by listing pros and cons of the DNA-free RNP method in plants.

- Pros:
  - Cas9 protein expression and sgRNA processing in vivo were not necessary;
  - no foreign DNA remained in the genome; and
  - homozygous mutants for multiple genes were generated at a single generation.
- Cons:
  - somaclonal variations possibly accompany during tissue culture;
  - the regeneration of whole plants from some crop protoplasts was difficult for some plants such as maize and soybean; and
  - safe and efficient delivery of RNPs should be developed.

### Next-generation waxy corn

Robert Meeley, Senior Research Scientist at Corteva Agriscience (DowDuPont), provided a second example of the application of genome editing in crop plants. He introduced his presentation on the “Next-generation waxy corn” by explaining that one of the main reasons for Corteva to work on waxy corn was met by this OECD conference: “The reason we are working on waxy corn is to have this conversation, because [we needed] to come forward with something that had a long history of safe use as a trait, [that] has important industrial uses both in food […], and in [other] industrial application, as well as ethanol.”

“We needed to make something quick and have this conversation now, in order to get it out on the market,” he added.

What I am hoping to convince you of today is that by putting together some very critical technologies and breakthrough technologies, and serial transformation in our stewardship practices, that we can get a product that will be accepted out there quickly, so that we can move the ball on the bigger goals that genome editing holds for us.

(Robert Meeley, “OECD Conference on Genome Editing: Applications in Agriculture—Implications for Health, Environment and Regulation”, 28–29 June 2018)

He stressed that the conversation on genome editing was all about listening to and engaging with stakeholders; the application of genome editing techniques was not set in stone, but subject to an ongoing dialogue.

Meeley described the characteristics of waxy corn, which had been known since 1908, commercially cultivated since the 1940s (as a tapioca substitute, due to its similar mouth-feel to that of the cassava plant extract), and sold by Pioneer since the 1980s. The current commercially available waxy corn was a recessive trait that was grown in an identity-preserved system: it was based on the deletion of 30 bp in the original “No. 2 Yellow Dent” corn, which turned the original dominant *Wx1* allele into a “null allele” (*wx1*) (i.e. a non-functioning gene) and thus increased the amylopectin content in the corn starch from 75 to 100% (Fan et al. 2009).^5^ He noted that the cultivation and processing of waxy corn was essentially a “closed loop”-system, providing a good risk-mitigation approach: the knockout crop was typically produced by growers and millers under contracts that stipulated a strict isolation during both the growth and the wet-milling process; 0.5% of US corn acres only were used for waxy corn production annually.

The contained production system of waxy corn made it an ideal candidate for a genome editing demonstration case: genome editing brought a significant advantage to the breeding process of waxy corn: while conventional breeding was conducted by the backcrossing of plants over a minimum of eight cycles to produce commercially hybrid seed lots (and could yield up to 10% of unintended content, despite the lengthy selection procedure), the CRISPR technology offered the recapitulation of a *Wx1* whole gene deletion in one dozen elite inbreds, using fewer breeding cycles and providing a higher precision in the edits compared to the conventional approach.

Corteva’s approach used two guide RNAs, designed to introduce cuts at the 5′ and 3′ ends of the gene, resulting in a 4 kb deletion. The company’s prime concern of this genome editing demonstration, however, was to convey a thoughtfulness and a correspondingly amended approach to stewardship, which consisted of a number of key-steps that pressure-tested a number of internal and external perceptions and relationships.

Highlights of the genome editing stewardship approach were:

- robust design, based on over 90 years of pedigree corn-breeding history (in the US), extensive knowledge of the germplasm, in-depth next generation sequencing routines, and the benefit of reference gene models that can be used to compare variations between haplotypes;
- elite transformation, conducted by producing somatic embryos through the co-bombardment of 12 inbreds with six different components (i.e. three CRISPR/Cas components and three helper components), using a gold-particle gun (Lowe et al. 2016, 2018);
- precise confirmation, yielding knowledge of the exact repair site variants across the inbreds, followed by a ‘Southern-by-Sequencing’ process, in order to confirm that no extraneous DNA was present (Zastrow-Hayes et al. 2015); and
- agronomic testing, demonstrating that the CRISPR-waxy corn out-yielded its conventional hybrids, while the starch obtained from it was indistinguishable from that obtained from conventional waxy corn.

Meeley concluded that the CRISPR technique represented a significant innovation to plant breeders; it generated products that could also be produced by spontaneous or induced mutagenesis deployed in traditional breeding, while no foreign DNA was present in the genome-edited end-product.

Corteva Agriscience confirmed that CRISPR-Cas Waxy Maize was produced without the creation of a novel combination of genetic material.

During the Q&A session following his presentation, he was challenged about which one of the two in-house genome editing technologies at Corteva (i.e. ZFN and CRISPR) would continue to be used. Meeley noted that there had not been a formal decision concerning this, but he suggested, that such decisions needed to take into account the regulatory discussion and the trait concept, and that considerations of cost and development procedures could foster the continued use of the ZFN technology.

### Crop plants with improved culture and quality traits

Peter Rogowsky from the French National Institute for Agricultural Research (ENS Lyon) delivered a presentation on “Crop plants with improved culture and quality traits for food, feed and other uses”, which was based on the “Genius” (Genome engineering improvement for useful plants of a sustainable agriculture) project, a research project funded with EURO 6 Million by the French National Research Agency, and conducted by a consortium of 10 public and four private laboratories over a duration of 7 years.^6^ The project focussed on nine cultivated plant species (i.e. four crop, two vegetable, one fruit, one forestry, one ornamental) and three model species (Nogúe et al. 2019).

Rogowsky alerted the audience to an important challenge posed by genome editing approaches: “When talking about gene editing, do not forget about the challenge to get the tool into the plant cell!” He added, “When you want to use genome editing on the large scale in agriculture, it has to work in elite genotypes, while most publications to date are applicable to lab genotypes only.” He pointed out that cellular engineering was still largely the bottleneck of genome editing, in part because it remained dependent on the species and genotype.

He showed the three methods of targeted gene modification, and illustrated their respective success rates in the species edited by the Genius project (see Table 2).

**Table 2:** The three ways to edit genomes used in the Genius project (after Peter Rogowsky, “Crop plants with improved culture and quality traits for food, feed and other uses”, 28. June 2018)

Genome editing type	Description	Successful establishment by Genius
SDN1 (site-directed nuclease 1)	Targeted mutagenesis yields a mutation at a predetermined site, but provided no influence on the type of mutation that occurred; this technique is typically used to create knockouts.	12/12
SDN2 (site-directed nuclease 2)	This ‘true’ genome editing allowed the control of both the site, where the edit is to take place, as well as the type of editing that should occur there. The efficiency of SDN2 is currently quite low; there are only a few examples of true SDN2 known to date.	2/12
Base editing	The base editing technique, which appeared over the past few years, is more limited than ‘true’ genome editing, because there are only certain types of edits that can be achieved with this technique. The efficiency, however, is as high as that of SND1.	2/12

Rogowsky presented a number of studies that had been conducted by Genius and discussed their outcomes:

- *Marker genes (basic research interest)*: Genius had demonstrated that visual marker systems were helpful to establish and optimise genome editing procedures. The project consortium had obtained marker genes through SDN1 and SDN2 modifications, yielding example traits, such as bleached leaves (in apple), yellow leaves (in rice and wheat), the loss of blue histochemical staining (in poplar), the loss of the purple colour (in tomato), and physcomitrella colonies that survive on 2-fluoroadenine, when they were mutated only.
- *Breeding tools*: Maize, as a representative of a haploid plant that rapidly produce pure homozygous plant lines, due to the asexual propagation of crops, had been modified using SDN1. The Genius consortium had thus demonstrated that modifications of plant reproduction presented an interest for plant breeding (Gilles et al. 2017).
- *Quality trait*: The Genius project had conducted an SDN1 editing approach on potato plants, in order to enhance the amylopectin content of the tuber to 100%, and through successful completion of the study, had been able to demonstrate that metabolic pathways provided numerous targets for genome editing (Andersson et al. 2017).
- *Flowering time*: Apple plants had been modified using SDN1, in order to achieve a shortening of the life of the perennial species; this could help to mitigate climate change and enhance crop rotation. Genius had been able to demonstrate that genome editing was a helpful tool for fast breeding.
- *Abiotic stress tolerance*: Rice had been subjected to SDN2 genome editing, in order to achieve tolerance to salinity in the plant (for rice cultivation in lower basins or on marginal land); this representative study of abiotic stress tolerance had validated the efficiency of cleavage and found that knock-in techniques without selective markers required an optimisation of all steps.
- *Disease resistance*: Tomato plants had been edited using SDN2, in order to yield resistance to potyviruses; the successful study had demonstrated that true genome editing (i.e. SDN2) was superior to the inactivation of host factors via knockout, which was known to affect the vegetative development of the plant.

Rogowsky went on to provide bibliographic information on targeted genome editing in crop plants to achieve improved nutritional components:

- *Low gluten wheat*: Two bread wheats and one durum wheat had been modified by SDN1 targeting 45 α-gliadin genes, in order to lower the gluten content of the grain, and thus provide a low-allergen food; the study demonstrated that genome editing could provide plant products with improved health characteristics (Sánchez-León et al. 2018).
- *Lycopene enriched tomato*: Multiplex targeted mutagenesis (SDN1) had been applied to yield a tomato with an elevated content of lycopene, an antioxidant that may prevent the onset of certain cancers. The research demonstrated the nutritional enhancement of a fruit that was an important part of human diet and a target for biofortification (Li et al. 2018).
- *High oleic acid soybean and camelina*: SDN1 had been used, in order to change the oil composition in soybean and camelina plants, with the aim to obtain longer shelf life without the use of artificial hydrogenation of the oil, and to also provide the additional health benefits of monounsaturated fatty acids. Although high levels of oleic acid could be found in other crops, the study demonstrated that modification of seed oil composition was a lever for improved food and feed (Haun et al. 2014; Morineau et al. 2017).

Rogowsky concluded that genome editing had become a major tool in basic research, and that it would have a significant impact on agriculture, as long as the following five conditions were fulfilled:

- the genotype and phenotype relationship of a plant was sufficiently known;
- there had to be a sharp increase in the efficiency of true genome editing (i.e. SDN2);
- breeding material (i.e. elite varieties) had to be accessible to those who conduct genome editing;
- SMEs (small and medium-sized emterprises) had to have access to genome editing (i.e. licensing should be possible at a reasonable cost); and
- genome editing had to be deregulated (compared to conventional transgenic approaches).

During a brief discussion following Rogowsky’s presentation, the possibility of deregulation of ‘real genome editing’ was debated: Would the regulator rely on a definition of a strict number of base-pairs that had been altered in the genome editing approach (for example: in order to distinguish between a deregulated SDN2 and a regulated ‘SDN3’)? It was clarified that the insertion of foreign DNA by genome editing techniques, using an SDN3 type approach, would probably fall in the scope of the GMO regulation in any case.

It was concluded that regulatory clarity was required, in order to further advance the field of genome editing research on commercially available crops: many positive effects were difficult to achieve by SDN1 (i.e. simply knocking out the gene, which often affected other traits than the targeted trait), making it necessary to get SDN2 to work in crop plants.

### Crop plants with enhanced disease resistance

Vladimir Nekrasov from the Plant Sciences Department at Rothamsted Research, the United Kingdom, provided the fifth contribution to the topic of “Applications of genome editing in crops”; in his presentation entitled “Genome editing as a tool for enhancing disease resistance in crops”, (Nekrasov 2019), he reminded the audience that the MIT Technology Review had voted the CRISPR genome editing technology one of the 10 breakthrough technologies in 2016.^7^ “By now,” Nekrasov added, “the technology has been applied in some model plants (incl. *Nicotiana, Arabidopsis*), and also in various crop plants (incl. tomato, barley, maize, brassica, wheat).”

In his opinion, this technology could be used in two different major ways. One of them was the use as an excellent tool for gene function studies in basic biology; a gene of interest was disrupted, in order to observe the resulting phenotype. Another use involved crop breeding, in which the technology posed an immense improvement, especially due to its ability to speed up the entire breeding cycle: instead of two sequential steps in the conventional breeding cycle (i.e. (i) parental cross of elite varieties, and (ii) intercrossing and/or backcrossing of breeding population), the technology enabled to introduce traits of interest into the elite variety in a single step without “linkage drag” (Scheben and Edwards 2017).

Nekrasov presented examples, in which genome editing techniques had been applied to engineer both recessive and dominant disease resistance in crops:

1. Recessive resistance could be achieved by elimination of susceptibility genes from the plant genome (example: resistance to *Xanthomonas oryzae* in rice (Li et al. 2012), and the mildew resistance in tomato plants (Nekrasov et al. 2017)).
2. Dominant resistance could be obtained by adding a resistance gene to the susceptible cultivar; this methodology essentially represented the inclusion of a transgene, or a gene that had been introgressed (example: improvement of disease resistance to geminiviruses in *Nicotiana* (Baltes et al. 2015; Chaparro-Garcia et al. 2015)).

Nekrasov illustrated the process of creating the mildew-resistant tomato: an optimised Cas9-sgRNAs-expressing vector was constructed using the Golden Gate cloning system, in order to target the susceptibility gene *SlMlo1*; tomato callus was transformed with the Cas9-sgRNAs vector and then targeted by Cas9 to induce a 48 bp deletion at the *SlMlo1* locus; T0 plantlets were regenerated to screen for homozygous mutants; and T1 seeds were backcrossed for segregation to obtain transgene-free T2 seeds. T-DNA-free homozygous *SlMlo1* tomato lines had been produced in less than 10 months, suggesting a high precision of CRISPR/Cas in tomato, confirming complete resistance to a mildew pathogen as well as verifying the targeted deletion and the absence of T-DNA and off-target mutations using whole genome sequencing.

Nekrasov pointed out, however, that the application of the CRISPR/Cas method to add a resistance gene was technically not a genome editing technique per se; the technique could rather be used to improve the disease resistance of a plant to DNA-viruses, by introgressing a transgene encoding Cas and guide RNA which subsequently targeted and destroyed the virus DNA, so that the CRISPR/Cas system essentially played the role of the resistance gene. Similar technology was used to create resistance to RNA viruses using Cas13a, an RNA-guided RNA nuclease, in plants. Since these methods relied on the insertion of a transgene, which induced the desired resistance, the resulting plant would probably be deemed a genetically modified (GM) plant.

During the Q&A session following Nekrasov’s presentation, the conference delegation discussed whether the insertion of a resistance gene would be deemed a gene engineering technique, even if a non-functioning homologue in the target crop was replaced by the functioning resistance gene in a wild relative using genome editing techniques. The delegation concluded that the regulatory decision would probably always come down to the question of how many changes had been introduced with a given technology. Nekrasov reminded the audience that several advisory bodies in Europe were of the view that SDN2 approaches that changed fewer than 20 bp should not be considered recombinant DNA, and should thus not be regarded as GMOs with regard to the Directive 2001/18/EC (Sprink et al. 2016; ZKBS 2012; EC 2001). The question remained, however, how the regulators would deem the introduction of 100 bp spread over more than five loci (i.e. with fewer than 20 bp each).

### Development of crop plants for international distribution

Petra Jorasch from the European Seed Association and the International Seed Federation provided the sixth and last contribution on the application of genome editing in crop plants. In her presentation entitled “The global need for plant breeding innovation”, Jorasch first outlined the vision and mission statements of the associations, thereby introducing the presentation’s core aim to explain the global need for plant breeding innovation (Jorasch 2019).

Jorasch outlined the plant breeders’ active response pledge to global challenges:

- withstand pests and diseases with fewer crop inputs;
- maximise resource use efficiency (water, land, nutrients); and
- stabilise and increase yields, despite climate change.

She noted how important it was to understand the long history of plant breeding, in order not to discuss the latest techniques of genome editing (e.g. CRISPR/Cas) in an isolated manner, because the entire field of plant breeding looked back on a long history of innovation. She went on to illustrate the milestones in plant breeding, spanning a timeline from 10,000 BC (when emmer wheat and (small) spelt wheat had been subjected to selection breeding) to the current time, where the field had now arrived at the milestone of “precision breeding”, which allowed an unprecedented specificity in plant breeding using genome editing and other breeding techniques. She pointed out that before the understanding and outlining of the “laws of genetics” by Mendel in 1866, plant breeding had essentially been selection breeding; only after the milestone introduced by Mendel had it been possible to intentionally cross plants to improve the offspring.

[Plant breeding innovation] is not about replacing one technology with another, but about adding to the toolbox, [so as] to increase the breeder’s choice to choose the right tool to address a specific problem.

(Petra Jorasch, “OECD Conference on Genome Editing: Applications in Agriculture—Implications for Health, Environment and Regulation”, 28–29 June 2018)

She explained that plant breeding innovation cycle, in which genome editing techniques also needed to be integrated, essentially consisted of two steps:

1. *Increasing genetic variations*: The main goal of the breeding process was the increase of genetic variations; it was typically achieved by either choosing the right germ plasm (i.e. old or new varieties, or plant genetic resources), or by use of the newer tools (e.g. random or targeted mutations, cisgenesis, transgenesis or epigenesis).
2. *Decreasing genetic variations*: The necessary second step in the plant breeding innovation cycle consisted in the selection of the plants that exhibited the desired phenotype, followed by (several years of) field trials, conducted in different environments.

This necessary two-step process demonstrated the reason why, for plant breeders, the application of genome editing techniques would still be subjected to a subsequent step of crossing, since the plants should grow in the field, and breeders needed to select the plants with the right characteristics. As previously described, in the case that a disease-resistant trait were to be introduced in a tomato plant, some populations might also exhibit some negative characteristics on other agricultural traits, and plant breeders would probably need to de-select such adverse outcomes during the selection step.

In the end, [plant] breeding takes place in the field; the variety has to perform in the field, and if it doesn’t perform, it will not become a new variety. We talk about ‘innovation in rubber boots’, because in the end, it’s the breeder in the rubber boots in the field who will select the right plants to get the new variety.

(Petra Jorasch, “OECD Conference on Genome Editing: Applications in Agriculture—Implications for Health, Environment and Regulation”, 28–29 June 2018)

Jorasch explained that genome editing made the breeding process a lot more efficient, while the main breeding goals did not change with the introduction of genome editing; Table 3 below listed many examples of possible successful applications of genome editing to plant breeding. Not all of the listed examples were necessarily on the market, but all of them were good examples of the techniques’ potential for innovation.

**Table 3:** Genome editing allows breeding goals to be achieved in a more efficient way (after Petra Jorasch, “The global need for plant breeding innovation”, 28. June 2018)

Quality traits	Yield	Resistance
Baking quality (e.g. *N*-glycans modification in barley, gluten free wheat) Brewing quality (e.g. low lox barley) Fatty acid composition (e.g. high oleic acid soybean/camelina; low sat. fatty acid canola) Increased vitamin content Improved shelf life (improved cold storage potato, non-browning mushroom/apple/potato) Starch quality (e.g. waxy corn, amylopectin potato, high-amylose rice) Food/feed quality (low-phytate maize, high fiber wheat)	Corn yield (pod shatter resistant oil seed rape, grain weight and enhanced grain number in rice, parthenocarpic tomato plants) Biomass yield (improved C3-carbon metabolism in df) Starch, protein, sugar, oil content (higher oil content camelina) Nutrient use efficiency Water use efficiency (drought tolerant soybeans)	Viruses (cucumis: zucchini yellow mosaic virus; papaya ring spot virus) Bacteria Insect Fungi (e.g. powdery mildew in wheat and tomato, late blight potato, blast resistant rice) Drought, heat, salt (salt stress tolerant rice, drought stress resistant corn) Herbicides (e.g. oilseed rape, linum, rice, potato)

She noted the “technique conundrum”, which described a contradiction found in the same product obtained using different breeding techniques. While on one hand the familiarity with a breeding technique increased with the time the technique had been used, on the other hand its specificity decreased. Even when four different plant breeding techniques (i.e. crossing & selection, random mutagenesis, rDNA, and genome editing) had been used to achieve the same product (e.g. waxy corn), our familiarity decreased with a decreasing age of a technique’s application in plant breeding (i.e. from crossing & selection to genome editing), although the specificity provided by the technique increased over the same sequence and in the same fashion.

We might run into a conflict between what we call the precautionary principle on one hand and the innovation principle on the other hand.

(Petra Jorasch, “OECD Conference on Genome Editing: Applications in Agriculture—Implications for Health, Environment and Regulation”, 28–29 June 2018)

“We need to find the right balance, and in the end it’s a compromise between both principles,” she added.

Jorasch advocated a consistent, science-based approach for regulatory oversight; the associations suggested the following underlying principle: “Plant varieties developed through the latest breeding methods should not be differentially regulated if they are similar or indistinguishable from varieties that could have been produced through earlier breeding methods.”

She summarised that plant breeders needed clear guidance on: (a) the scope of regulatory oversight; and (b) the corresponding timelines and requirements, as well as consideration of the existing regulatory mechanisms. In order to illustrate the need for a global approach, she described the typical movement of vegetable seeds around the world: between the original breeding of the parental lines (in the Netherlands), and the arrival of the seed at its final market (in Mexico and Brazil), the seeds had been processed in seven different countries around the world. This scenario alerted to the potential societal and economic risks that a non-harmonised system of patchwork regulations and asynchronous decisions would pose.

During a brief Q&A session, the delegation discussed the potential impact that genome editing and its regulation may have on intellectual property rights: it was highlighted that the plant breeding community was already committed to using patent protection not to enable monopolies, but to create return on investment on one hand, and to allow access to the technology or the germ plasm to other innovators on the other hand; this was specifically reflected by the breeding rights laid down in the patent system.

### Genome editing in chicken

Mark Tizard from the Australian Animal Health Laboratory, Commonwealth Scientific and Industrial Research Organisation, Australia, provided the second contribution to the discussion of genome editing applications in farm animals (Tizard et al. 2019); in his presentation entitled “Genome Editing in Poultry: Opportunities and Impacts”, Tizard first outlined the importance of poultry to global food security: as a major source of protein, poultry meat was predicted to become the single most consumed meat by 2020, while the eggs produced by poultry were another major reason for its importance to food security.

The impact of genomics on poultry farming had already been substantial, Tizard reported. Poultry farming had initially relied on selective breeding, and was later assisted by genomic markers, as the chicken genome sequence had become available. He pointed out, however, that improved intensive production could increase health and welfare issues, and that some key traits in poultry were too complex to tackle with selective breeding approaches. In this context, genome editing was set to provide a number of game changing interventions, such as the fixation of valued alleles, the deletion of detrimental alleles, and the allele transplantation between strains (within species).

Tizard noted, however, that possible “off-target” issue still posed a major caveat to a full adoption of genome editing techniques in poultry farming. He went on to explain that precision genome engineering in birds was very difficult (i.e. in birds, the single cell zygote was intimately linked with the yolk, rendering it almost impossible to manipulate). A different approach was therefore required to make genome editing available to poultry: primordial germ cells (PGCs) (i.e. progenitors to ovum- and sperm-forming cells) were accessible either outside or inside of the growing chick embryo; these cells could be retrieved, cultured and manipulated in vitro. He pointed out, however, that the handling of PGCs outside the bird was very difficult. Tizard’s team had developed an easier approach to directly inject the PCGs with genome editing tools in vivo*/*in ovo. They explored a more accessible approach, so-called sperm transfection assisted gene editing (STAGE), involving transfecting sperm with genome editing tools and then using the sperm for artificial insemination. Compared to the methods using PGCs, STAGE provided the largest improvement of speed in the breeding cycle, because it did not necessitate the creation of chimera, and could thus yield edited hens and roosters within a single year (i.e. after ca. 6 months of heterozygous offspring rearing and ca. 6 months of homozygous offspring rearing).

CSIRO is now using CRISPR, because it is the most efficient way forward.

(Mark Tizard, “OECD Conference on Genome Editing: Applications in Agriculture—Implications for Health, Environment and Regulation”, 28–29 June 2018)

Tizard noted that examples of the application of genome editing in chicken included the following:

- *Yield improvements to the vaccine industry*: CSIRO had launched a spin-out company, which provided a significant innovative improvement to the vaccine industry: while two chicken eggs were traditionally required to make a single dose of vaccine, a single egg laid by the genome-edited chickens could generate five doses of vaccine.
- *Disease resilience*: Genomics and genome editing were revealing opportunities to enhance resilience to important diseases such as avian influenza: disease impacts on food production and human health and safety could be reduced.
- *Allergen*-*reduced eggs*: Genome editing could remove the allergen ovomucoid (Ovm) from chicken eggs. Ovm was the most allergenic egg white protein, but it represented only 10% of the total egg white protein, and played no clear role in fertility, egg formation or nutrition. While the resulting eggs could not be called “allergen-free” because other allergens remained in the egg, most egg-allergy sufferers could eat eggs, in which Ovm only had been removed.
- *Selective hatching*: Eggs containing male chicks could be deselected from the hatching process by placing a marker on the male-determining chromosome, in order to provide an alternative to culling day-old male chicks, because they were not efficient to farm (based on their inability to lay eggs and their inferior meat).^8^

Tizard concluded that genome editing techniques already allowed the creation of precise, targeted modifications to the chicken genome. CSIRO’s hope was that the use of the technology would soon: (a) improve the cost and efficiency of production (through improved sustainability, health and welfare), (b) improve food safety, and (c) enhance vaccine production.

He pointed out, however, that all of the positive impacts of genome editing relied on a number of important prerequisites concerning genome editing:

- safety data combined with effective regulation of the technology;
- public understanding and attitudes to genome editing (cf. GMO); and
- how these factors had an impact on industry adoption.

Tizard noted how important it was for the advancement of genome editing to have scientists, policy-makers and regulators in one room together, in order to have the discussion that was the focus of the current OECD event.

### Application of genome editing in cattle

Alison Van Eenennaam from the Department of Animal Science at the University of California, Davis, the United States, delivered the third conference contribution on the topic of the application of genome editing in farm animal (Van Eenennaam 2019). She commenced by highlighting the significant role that cattle played for food security, as both the animal’s meat and its milk were an important source of protein. In addition, the ruminants turned inedible forage into edible protein. Milk and meat from cattle and buffaloes made up 45% of the global animal protein supply, and it had already been forecast that the demand for animal-derived food could be 70% higher in 2050 than it had been in 2005 (FAO 2018; Alexandratos and Bruinsma 2012). This forecast necessitated an acceleration in the rate of genetic gain in global cattle, because too many countries currently suffered from low productivity of their dairy industries.

Van Eenennaam explained that the rate of genetic gain essentially depended upon four components in the breeders’ equation:

$$ Genetic\; Change\; per\; year = \frac{{Reliability \times Intensity \times \sqrt {Genetic \;variation} }}{Generation \;Interval} $$

where: Reliability = accuracy of selection (i.e. the accuracy of the breeding value prediction); Intensity = selection intensity (i.e. a measure of accuracy of selection decisions); Genetic variation = the genetic material, with which breeders can work; Generation interval = time between generations.

The genetic variation was the one element in the equation, at which breeders were a bit stuck, and where genome editing could provide one of the biggest advantages to cattle breeding, she noted.

Van Eenennaam explained that genome editing in cattle could be utilised either in a somatic cell nuclear transfer (SCNT) approach, or in a microinjection approach; in both cases, the edited embryo would be transferred into the mother cow prior to a natural delivery.

Genome editing in and of itself is the cherry in the breeders’ sundae.

(Alison Van Eenennaam, “OECD Conference on Genome Editing: Applications in Agriculture—Implications for Health, Environment and Regulation”, 28–29 June 2018)

“[Genome editing] will be able to introduce useful alleles without linkage drag, and potentially bring in useful novel variation from other species,” she added.

Van Eenennaam listed a number of possible applications of genome editing in cattle farming, and then focussed on discussing one specific problem that the technique could help solve: the removal of horns from dairy calves to protect human handlers and other animals. The removal was traditionally achieved by using a procedure known as “disbudding”, in which local anaesthetic was administered before an electric disbudding instrument was applied to the hornbud, where it provided heat to kill the horn-growing cells. Hornless dairy sires (three in Jersey cattle and 19 in Holstein cattle)^9^ had inferior genetic merit, rendering selective cross-breeding useless to obtain the desired hornless dairy cows, but genome editing could provide a route to introduce the hornless allele of the Angus cattle into Holstein dairy cows. The corresponding TALEN approach, conducted in 2015, represented an intra-species allele substitution between a dominant Angus allele of 212 bp and a recessive Holstein allele of 10 bp. The genome editing had been followed by SCNT cloning and embryo transfer, resulting in two healthy bull calves. Semen collected from one of the bulls confirmed that he was homozygous polled (PP) and fertile in vitro; straws were subsequently frozen for artificial in vivo insemination. Five healthy bull calves and one heifer calf had been subsequently born in 2017, all of them hornless (i.e. heterozygous Pp for polled).

Other examples of the successful application of genome editing in cattle included the following:

- zygote injection of TALEN mRNA to target the myostatin gene, in order to obtain double muscle Nellore cattle (Proudfoot et al. 2015);
- precise zygote-mediated genome editing using the TALEN technique to eliminate beta-lactoglobulin (BLG), a major allergen in cows’ milk (Wei et al. 2018); and
- reduction of the susceptibility to tuberculosis in cattle using a single Cas9 nickase to insert the natural resistance-associated macrophage protein-1 (NRAMP1) gene through SCNT (Gao et al. 2017).

Van Eenennaam summarised that, by permanent and cumulative genetic improvement, genome editing provided an important potential solution to replace the treatment of animal diseases using antibiotics or chemicals. She noted, however, that regulatory hurdles currently proved detrimental to the advanced use of the technique in cattle farming:

In January 2017, FDA draft guidance considered all gene edited animals whose genome have been ‘altered intentionally’ to be drugs.

(Alison Van Eenennaam, “OECD Conference on Genome Editing: Applications in Agriculture—Implications for Health, Environment and Regulation”, 28–29 June 2018)

She questioned if it made sense to regulate polled dairy calves differently from polled beef calves (Van Eenennaam 2018; Carroll et al. 2016), and pointed out that Canada had meanwhile moved to a novel product-based regulation, and thus deregulated an alliance between Semex, a farmer-owned cattle genetics organisation and Recombinetics, a genome editing company, in order to implement a precision breeding programme to introduce the hornless trait into elite dairy cattle using genome editing.^10^

During the brief Q&A session following Van Eenennaam’s presentation, the delegation concluded that whatever regulatory framework might be given to genome editing, it had to fit with cattle breeding practices, because existing and novel techniques would be used in a complementary, not an exclusive, fashion. In cattle, genome editing would add an extra two months to the breeding cycle (i.e. results would have to be test-driven according to the desired phenotype), before it could be introduced into the breeding cycle. Van Eenennaam pointed out that this was a reasonable approach, while it was otherwise impossible to slow down the annual breeding rate (e.g. for a multi-year evaluation), because most of the value of cattle breeding was concentrated in the established “breeders’ sundae”.

### Farm animals in aquatic systems

Anna Troedsson-Wargelius from the Molecular Biology Section of the Institute for Marine Research, Norway delivered the final contribution on the topic of applications of genome editing in farm animals; in a presentation entitled “Farm animals in aquatic systems” Troedsson-Wargelius pointed out the importance of aquacultures to global food security (Wargelius 2019): in 2016, 171 million tonnes of fish had been produced, of which 47% came from aquaculture (FAO 2018). In Norway, the world’s largest producer of Atlantic salmon, the aquaculture industry represented the country’s second largest economic sector. The farming of Atlantic salmon in aquaculture had grown sixfold since the 1990s, but had levelled off in recent years, due to reasons of sustainability and a governmental cap on further expansion.

The main sustainability challenges were:

- diseases (such as “salmon lice”/“sea lice”);
- genetic introgression of farmed fish into wild populations; and
- feed resources (i.e. the optimisation of the farmed fish’s filet quality).

Troedsson-Wargelius noted that one major challenge affecting breeding innovations in salmon cultures was the long breeding time: male salmon could sometimes be brought to breed after 1 year, but female salmon did not do so until after 3–4 years.

Genome editing with the ZFN editor had already been tried without much success; encouraged by the successful application of the CRISPR/Cas technique in other fish species (i.e. tilapia, catfish, carp, and rainbow trout (in vitro)), Troedsson-Wargelius’ team was now testing the editor as a research tool and for possible industrial applications, in order to tackle the following challenges in salmon aquacultures:

- *Sterility of aquaculture* (i.e. the genetic containment of wild populations): A single fish-farm container could contain up to 200,000 fish, whose escape into the wild would be ecologically disastrous. While there were other ways than genome editing to render salmon sterile, most of those had severe side-effects. Under the project “Salmosterile”, Troedsson-Wargelius’ group had used the CRISPR/Cas9 editor to knockout *dnd* (i.e. a factor required for the survival of germ cells in vertebrates) and the tracer *alb* (i.e. absence of *alb* resulted in complete loss of pigmentations in salmon), in order to facilitate the subsequent study of the mutant animals through their long generation times. The group had found a high correlation of induced mutations for the target and the tracer, and had thus been able to report the first successful knockout study of *dnd* in fish, leading to complete loss of germ cells in the F0 generation (Wargelius et al. 2016). The project continued and was now following the salmon for an entire life cycle (2017-2020) (Kleppe et al. 2017).
- *Filet quality* (i.e. the analysis of synthesis pathway of omega-3 fatty acid to improve nutritional composition): Troedsson-Wargelius’s group had studied the knockout of *elovl2* that encodes the enzyme involved in the elongation of the omega-3 fatty acids; combined with the knockout of the *alb* tracer, the group had been able to demonstrate positive results and establish a methodology to better study the effect of different feed varieties on farmed salmon populations.
- *Controlling age at maturity*: A sequencing study of wild salmon in two different areas had identified a locus controlling the salmons’ sea age at maturity (Barson et al. 2015). Troedsson-Wargelius’s group was currently working on introducing the relevant single nucleotide polymorphisms (SNPs) into other (farmed) salmon, in order to verify the effect of introduced SNPs on age at maturity (Ayllon et al. 2015).
- *Disease resistance*: A whole genome sequencing study of eight populations of wild salmon had identified immune-related genes that might be involved in disease susceptibility (Kjærner-Semb et al. 2016). Troedsson-Wargelius’ team is working on these genes to introduce the SNPs found in these genes through HDR, in order to elucidate the involvement of the genes in disease resistance.

During her concluding remarks, Troedsson-Wargelius pointed out the importance of Responsible Research and Innovation (RRI) practices to her group’s work: these practices included regular interactions with the public, the media and the expert community through pro-active communication, stakeholder surveys, in-depth reviews of ethics and integrity of projects, and the integration of local high schools.

## Panel discussion session 1: Applications of genome editing in agricultures

Amongst other things, the 1-h long panel discussion, which included all speakers and the moderator of Session 1, aimed to summarise the types of current and proposed applications of genome editing for the development of agricultural products and the techniques being used, highlighting the similarities and/or differences between applications and products in plants and animals, and any opportunities and challenges associated with various techniques and types of applications. The panel had been asked to also consider the realistic prospects for the various applications of genome editing in agriculture: which applications were available in the near-, medium- and long-term future?

### Science, technology and policy challenges to genome editing

The conference delegation engaged all Session 1 speakers in a lively, challenging debate: when asked, what improvement the researchers wished for the most, in order to advance their application of genome editing techniques, the scientists listed the following priorities:

- multi-gene insertion in plants (to solve certain complex diseases);
- improved base editing enabling any substitution of a nucleotide/multiplex base editing in plants;
- better crop sequencing (including minor crops)/improved understanding of genotype–phenotype relations, in order to aid the discovery of beneficial alleles;
- improvement of the delivery approach into the nucleus for DNA-free genome editing in plants;
- genome editing without tissue culture, in order to overcome the problems posed by plant regeneration;
- easy genome editing by introducing Cas nucleases into pollen or sperm cells in plants;
- improved HDR (homology-directed repair) tools to knock-in point mutations in plants and animals;
- dCas9-fused recombinase that allows DNA insertion at a specific site in animal genomes;
- access to genome editing (and other GM tools in general) for animal breeding programmes;
- availability of in vitro tools (including in vitro spermatogenesis) as essential tools for animal breeding;
- improved Cas proteins for aquaculture;
- a large scale seed production system; and
- legal clarity and certainty/the use of genetically engineered (GE) animals, in order to really bring this innovation to the market.

In the future, one will hopefully be able to create the required chromosomes in vitro and use those in genome editing.

(Panel discussion, “OECD Conference on Genome Editing: Applications in Agriculture—Implications for Health, Environment and Regulation”, 28–29 June 2018)

### Challenges to the public acceptance of genome editing

During a lively debate, both the panel and the audience stressed how important it was to secure the public acceptance of genome editing; this was specifically important in the case of animals, which people tended to identify with more than with plants. It was noted, however, that not only laypeople and consumers needed convincing of the benefits of genome editing, but that engagement efforts should include food producers (e.g. farmers), too.

There will be [genome editing applications] that industry will pick, because they are of value to their processes, but in the end, it needs to be accepted by the public.

(Panel discussion, “OECD Conference on Genome Editing: Applications in Agriculture—Implications for Health, Environment and Regulation”, 28–29 June 2018)

Policy makers and regulators carried a major part of the responsibility to foster a successful implementation of genome editing across different application, because the final stage of any public acceptance was always the translation of its benefits to the public. It was pointed out, however, that the public acceptance of genome-edited products did not follow a one-size-fits-all approach, because it depended on the overall socio-political setting in a given society and country (e.g. food security could be a very relevant aspect in some countries, while it played little role in others). In Europe, the emphasis of “sustainable agriculture” was currently regarded as the most important criterion. Industry and policy makers needed to communicate better regarding the prevalent aims to meet these public needs and challenges, and acknowledge the differences in ethical opinions between countries, ethnicities and religions.

The delegation was alerted not to over-promise the technology; while techniques like SDN2 had not been fully mastered, it would be foolish to overhype genome technology with results based on the technique.

Other important factors to secure the advancement and application of genome editing in agriculture included the following:

- Regulators should focus on the endpoint of the products instead of the technology.
- Scientists and companies should communicate the goals that are to be achieved by application of the technology (e.g. animal welfare).
- Policy makers and regulators should foster global cooperation, in order to establish a consensus.
- Scientists and decision makers should precisely explain what genome editing could do and what it could not do.
- Agriculture companies should provide farmers with information about their products and guidance on how to use them.

### Specific ethical challenges

When asked about the ethical limits to what scientist could and should do, the experts stressed that even in the absence of a formal oath for researchers, the credo “do no harm” represented a principal rule for them: in the case of farm animal research, studies were always based on the expressed fulfilment of a criterion to improve animal welfare. Special ethical considerations applied to the field of medical research, however, because here, disease-carrying animals had to be deliberately created in order to find treatments and cures for human patients.

### Technology assessment based on costs and benefits

It was agreed that innovations and advances in conventional breeding methodologies should always be considered alongside the evaluation of novel techniques, such as genome editing: based on the example of improving the productivity of dairy cattle in Sub-Sahara Africa, the panel experts pointed out that some approaches to better adapt animals to diseases and other possible challenges were as simple as changing the animal feed, while genome editing was a tool that could help with the otherwise impossible goal of introducing genetic heat tolerance.

The delegation agreed that balanced open dialogue processes should always address both the responsibility for what was done and the responsibility for what was not done.

### Food safety challenges

The expert panel pointed out that even the most advanced genome editing techniques could probably not achieve a world of entirely pathogen–free animals and plants, because in the race between pathogens, hosts and remedy, the former would never fully disappear.

For issues of food safety, gene editing is secondary to gene discovery.

(Panel discussion, “OECD Conference on Genome Editing: Applications in Agriculture—Implications for Health, Environment and Regulation”, 28–29 June 2018)

### Summary

The panel summarised its discussion:

- Genome editing could not be assessed with a view to its potential risks alone; both the costs and the benefits of everything it entails had to be discussed.
- Any assessment needed to meet the right balance between innovation and precaution, while not over-hyping or over-estimating one or the other.
- More funding should be made available for (basic) R&D of genome editing, if the technology’s benefits were to be secured.
- Case-by-case analyses of both the risks and the benefits of genome editing were currently still necessary, because the traits that could be achieved with genome editing were very diverse, and the respective risks associated with these traits were very different.

## Session 2: Risk and safety considerations

This session explored any risk and safety considerations that may be associated with the application of genome editing techniques in agriculture. It recapitulated the evolution and the state of risk/safety assessment for the products of modern biotechnology, both for food/feed and environmental assessments. The session considered the application of genome editing techniques, the nature and specificity of the genetic changes they created, as well as the specificity of targeted changes and evidence about the nature of any off-target changes.

Presentations and discussions during this session comprised a comparison with the body of knowledge of genetic changes (intended and unintended) from other approaches, such as conventional breeding, radiation or chemical mutagenesis, and “classical” genetic modification/engineering. Each presentation highlighted the practical approaches to identifying any hazards and addressing any potential risks, including the methods applied and the information needed.

The session was introduced and moderated by Huw Jones of the Institute of Biological, Environmental and Rural Sciences, at Aberystwyth University in the United Kingdom (Jones 2019).

### Current risk assessment approaches for environmental and food and feed safety assessment

Jeffrey Wolt from the Biosafety Institute for Genetically Modified Agricultural Products, Iowa State University, United States, provided an overview of the history of development and the current application of risk and safety assessment approaches to agricultural products including genetically engineered/modified organisms (GEOs/GMOs) with a variety of introduced traits (Wolt 2019).

#### Current risk and safety assessment approaches and their development

Wolt laid the basis for his presentation by listing a number of “key foundational documents” that had been developed within the past 32 years, during the time when the first recombinant DNA-derived organisms moved out of the laboratory and greenhouses into small-scale field trials and subsequently into wide-scale release. “These documents are found to be ‘foundational’ in terms of setting the paradigm for risk assessment (in this area) that we continue to use with regard to both environmental release and food and feed safety,” he noted. Wolt furthermore pointed out a number of “technical information documents” that had been developed subsequent to the key foundational documents.

Key foundational documents:

- OECD (1986)—the ‘Blue Book’: “Recombinant DNA Safety Considerations. Safety considerations for industrial, agricultural and environmental applications of organisms derived by recombinant DNA techniques (“The Blue Book”)” (OECD 1986);
- WHO (1991): “Strategies for assessing the safety of foods produced by biotechnology—Report of a Joint FAO/WHO Consultation” (WHO 1991);
- OECD (1993a): “Safety considerations for biotechnology: scale -up of crop plants” (OECD 1993a);
- OECD (1993b): “Safety evaluation of foods derived by modern biotechnology: concepts and principles” (OECD 1993b);
- OECD (1993c): “Traditional crop breeding practices” (OECD 1993c); and
- WHO (2000): “Safety aspects of genetically modified foods of plant origin—Report of a Joint FAO/WHO Expert Consultation on Foods Derived from Biotechnology” (WHO 2000).

Technical information documents

- CAC (2003a): “Codex principles and guidelines on food derived from biotechnology” (CAC 2003a);
- CAC (2003b): “Guideline for the conduct of food safety assessment of foods derived from recombinant-DNA plants” (CAC 2003b);
- OECD (since 2006): Consensus Documents in the series on “Harmonisation of Regulatory Oversight in Biotechnology”^11^;
- OECD (since 2002) Consensus Documents in the series on “Safety of Novel Foods and Feeds” (incl. sub-series on ‘plants’ and ‘mushrooms’)^12^; and
- CBD (2016) “Guidance on risk assessment of living modified organisms and monitoring in the context of risk assessment” (CBD 2016).

Wolt noted that—despite its age—the OECD Blue Book was still highly relevant to the risk assessment of modified plants today; he specifically pointed out the relevance of several observations made in the Blue Book (OECD (1986)):

- the risk assessment for rDNA-derived organisms was in principle no different than those for traits developed through traditional breeding;
- the safety assessment of any new organism relied on the knowledge of the parent and differences from the parent, regardless of the way employed to derive it; and
- there was no scientific basis for specific legislation for the implementation of rDNA techniques and applications.

He concluded that the last point might need to be reconsidered in the light of genome editing.

Wolt listed the underlying scientific principles of risk assessment that were further elaborated in the OECD Blue Book (OECD (1986)), and concluded that the foundational paradigm for risk and safety assessment proceeded from an established baseline, which consisted of the characterisation of: (a) the organism and its environmental release (in a manner amenable to probabilistic approaches), (b) its establishment and persistence in the environment, and (c) the resulting human and ecological effects (analysed—in principle—through existing methods).

#### Current applications of risk and safety assessment approaches

Wolt pointed out that the key foundational documents essentially elaborated a “comparative risk assessment” between the novel food or feed and an established food or feed that was familiar and/or had a history of safe use. Part of this comparison was the understanding of an “incremental risk”, which was established relative to a baseline of traditional plant breeding. He emphasised the concept of “substantial equivalence”, which defined one of the key elements of the comparative risk assessment: it did not represent a safety assessment per se, but a method for hazard identification to determine the needs of a safety assessment. Based on the paradigm, risk assessment had proceeded: from the concept of familiarity; to identify incremental risk; in case-by-case; in a stepwise approach; and reflecting the knowledge of relevant information on the crop, traits and the environment.

He summarised that the OECD, WHO and Codex documents together provided a consistent, powerful and sound risk assessment and safety assessment paradigm that still stood the test of time; moreover, the OECD Consensus Documents had helped to improve the risk assessment and safety assessment. He pointed out, however, that the implementation of the paradigm within various regulatory regimes had often departed from the spirit of the guidance.

We want to use science-based principles to understand the risk and safety of novel food and feed products,” Wolt said, “but our science is employed within […] social, legal and political constraints within given regulatory regimes, so that [….] what we see is a spiralling study complexity with a technology, which seems to be unrelated to the probable harm associated with that technology.

Regulators, scientists and developers all played a role in the increasing study complexity, because the simple conduct of their day-to-day jobs sometimes led to a cross-fertilisation between the roles, and thus a convolution of the resulting study designs and approaches. A good example of this sequence of events was the “problem of 2^nd^ order risk”, which could be rephrased as “the risk of the risk/safety assessment being wrong”, manifesting itself either by missing a significant risk, or by overanalysing a non-significant risk.

Current knowledge suggests over-analysis of non-significant risks for GEOs/GMOs.

(Jeffrey D Wolt, “OECD Conference on Genome Editing: Applications in Agriculture—Implications for Health, Environment and Regulation”, 28–29 June 2018)

Wolt pointed out that the risk and safety assessment community now looked upon “reasonable certainty of no harm from more than two decades of environmental deployment and occurrences in foods/feeds.”

#### Implication for the risk and safety assessment of genome-edited plants

Wolt highlighted the probable assumptions that the risk and safety assessment of genome editing derived from novel foods and feeds could be based on:

- Deep experience with assessing traditional, mutational and molecular breeding led to the conclusion that there were no new risks.
- Substantial interesting scientific questions were raised, for example, regarding improved efficiency and specificity or targeted mutations; it needed to be noted however, that these “nice to know” questions should not translate directly under a regulatory need, while regulatory questions only demanded “need to know” answers.
- At this stage of the development of genome editing technology, risk management could be more effective than risk assessment (i.e. science governance mechanisms could assure best practices in genome editing study designs).
- The conflation of risk management with risk assessment had to be avoided.

Based on above observations, Wolt recommended implementing sound problem formulation:

- Clear problem formulation was a priority, in order to guide the need for, and nature of, the risk and safety assessment that was to be conducted.
- The risk analysis framework needed to remain flexible, in order: (a) to accommodate new scientific understanding and risk analysis frameworks, and (b) to enable its use as a “process map”, rather than a list of studies.
- Genome-wide assessments as risk assessment paradigms should be avoided.
- The “process—product conundrum” might need to be reconsidered; while scientific risk and safety assessment for novel food and feed was product-focussed, regulatory constraints could argue that a process-focussed approach was necessary.

During the Q&A session following Wolt’s presentation, it was noted that the identification and understanding of off-target effects of genome editing were valid research topics, but that they did not represent risk assessments.

Challenged as to whether the rapidity and widespread applicability of genome editing techniques did not constitute a risk that was specific to the technology, Wolt noted that such characteristics were, indeed, pathways to risk, but that the nature of the risk was still the same.

The discussion concluded that unintended effects of genetic engineering/modifications in crops had been overestimated in the past, and that the higher precision promised by genome editing provided the community with the opportunity to focus on the risks of intended effects, as long as the genome editing tools were adequately improved.

### Genetic variations and potential risks: traditional breeding and genome editing

Yutaka Tabei from the Institute of Agrobiological Sciences, National Agriculture and Food Research Organization, Japan provided the second contribution to the session on “Risk and safety considerations” (Tabei 2019): he reflected on intentionally and unintentionally introduced genetic variations found in the products obtained by traditional breeding, genome editing, and conventional genetic modification/engineering to compare the types and numbers of genetic changes arising from these breeding methods. He quantitatively evaluated the differences and similarities of the mutations between breeding methods based on the whole-genome sequence data.

Tabei based his presentation on the Japanese National Programme entitled “Cross-Ministerial Strategic Innovation Promotion Program” (SIP), which aimed to develop new products and materials using genome editing and their social implementation. This programme consisted of the following four thematic groups:

1. Next-generation breeding technology consortium
2. Omics breeding technology consortium
3. Genome editing breeding consortium
4. Social implementation consortium

Tabei highlighted the activities of the social implementation consortium, which included accumulation of scientific data to promote public understanding of new breeding techniques. First, he reported the result of a public survey on genome editing and other new breeding techniques: some respondents expected much from genome editing, and others agreed to the use of genome editing if it could lead to the breeding of beneficial crops, but many respondents could not judge. While they understood the benefits of using these new breeding techniques, they still had concerns about the impacts of these breeding techniques on food safety and the environment. Furthermore, there were some responses suspecting the complete elimination of foreign DNA and the existence of unintended modifications such as off-target mutations.

Tabei then presented the result of scientific studies conducted to address these concerns. The studies involved: (a) the comparison of the occurrence of mutations among different breeding processes including tissue culture, transformation and genome editing, and (b) the detection of a small DNA fragment inserted in the genome. Rice was used as a model plant, because rice was the most important crop in Japan. The programme aimed to identify basic information on the mutations induced by each breeding technique, as well as on the detection of short DNA insertions, in order to use the data thus obtained for science communication and other activities.

The SIP programme described by Tabei had been able to establish the following results:

- Mutations caused by genetic engineering and genome editing in rice fell within the range of the somaclonal variation generated by tissue culture, and also within the variation observed in different geographic strains of the same cultivar.
- Foreign gene insertion of as short as 20 bp could clearly be detected in the whole genome sequence using a small fragment pattern matching method, which was already used for some plants such as rice, broccoli, wheat and tomato, in order to confirm that the genome-edited progeny were null-segregants.

He noted the possibility that somaclonal variation was caused by genetic stresses, which was known to induce transposition of transposons. In order to avoid unintended modifications in genome-edited products, his group introduced a method in which tissue culture was not involved; iPB (i.e. in planta particle bombardment) methods could deliver genome editing tools in the form of RNA, protein or RNP to the apical meristem cells. This DNA-free editing was successfully used in many plant species, such as wheat, to reduce somaclonal variation and to be efficient genome editing tools without unintended integration of foreign DNA.

### Considerations of unintended effects in genome editing applications

Marie-Bérengère Troadec of the Scientific Committee of the High Council for Biotechnology of France, presented the council’s opinion regarding unintended effects in genome editing applications (Troadec et al. 2019). Recognising that all breeding techniques had the potential to cause unintended phenotypes, this presentation explored safety considerations arising from unintended or off-target effects in genome editing applications, including the frequency and detection of off-target effects, and how these compared to other breeding techniques during the process of product development.

Troadec pointed out that her presentation was based on the opinion of the French High Council for Biotechnology (HCB), which was an independent body in charge of advising the French government on issues relating to GMOs and other types of biotechnology, but that it did not reflect the position of the French government. She referred the conference delegation to the recently published HCB “Opinion on New Plant Breeding Techniques”, which included genome editing techniques such as CRISPR; it had been developed and published in 2017 by request of the French Minister of Agriculture and the Minister of Environment (HCB 2017).

She stressed that any consideration of genome editing should also always look at other gene engineering/manipulation techniques; in this light, the innovation provided by genome editing could be more clearly understood:

- *Mutagenesis*: Traditional methods of mutagenesis relied on random mutations that had been induced by physical means (e.g. gamma irradiation), chemical treatment (i.e. base-analog, alkylating agents, or intercalating agents), or by biological means (e.g. transposon-mediated). Genome editing enabled a site-directed mutagenesis, based on the SDN1 technique.
- *Transgenesis*: Genome editing offered the SDN3 approach (i.e. a site-directed insertion of a foreign gene) to develop a new crop.

All genome editing approaches were based on three elemental steps: 1. Targeting, 2. Cutting, 3. Repair. SDN1 techniques allowed the host to conduct an unbiased, random DNA repair, while SDN2 and SDN3 techniques provided repair templates, which prescribed the exact sequence, with which the cut was to be filled: an allele-conversion (in the case of SDN2), or targeted integration of a sequence of interest (in the case of SDN3). Genome editing furthermore provided the possibility to carry out the full targeting-cutting-repair-sequence at several target sites simultaneously (i.e. a process called “multiplexing”).

Troadec went on to address the potential unintended effects that genome editing could cause at three different levels:

1. *Unintended effects at the level of the genome or the cell*: At this level, the HCB had identified two types of possible risks:
  - Unintended effects *due to the persistence of the effectors* (i.e. the Cas9 nuclease (DNA, RNA, protein) and the guide RNA (DNA, RNA)): The HCB recognised that the presence of guide RNA and nuclease could indeed induce off-target cuts or genetic modifications, and thus recommended that the absence of effector-coding genes be verified. Moreover, the HCB concluded that, if effector genes were present and stably transmitted in a plant, the resulting plant would be transgenic and thus fall under the EU GMO regulation.
  - Unintended effects *due to off*-*target modifications*: This form of genome modifications was essentially any form of modification that was different from the one intended by the genome editing approach. The HCB noted that off-target modifications necessarily implied not only the off-target effects resulted, but also the off-target mutations induced by SDN2 and SDN3; which would probably be the same as those by SDN1, since the probability of recombination of template DNA with the genome at the off-target site was very small and negligible. It thus seemed that off-target modifications occurred at some limited number of sites that were homologous to the guide RNA sequence (with up to five mismatches), and that they could not be easily distinguished from the natural mutations found in plants. The HCB nevertheless noted that a backcrossing step during the selection process following genome editing could eliminate off-target modifications. In order to prevent off-target modifications all together, the HCB suggested optimising the choice of Cas9 and guide RNA (and its specificity), but admitted that such optimisation required a good knowledge of the plant genome (and not just the model variety of the species). The Council discussed the possibility of sequencing putative off-target regions (known from in silico predictions) as a routine plant analysis for a few years, in order to collect data on the frequency of off-target cuts, however, a major difficulty lay in the availability of the full genome sequence of the variety used for breeding.
2. *Unintended effects at the level of the trait* (i.e. the plant): The HCB found no putative risks specific to genome editing at this level. Both direct and indirect common risks arising from the desired traits were different for each individual trait and were not affected by the technique used to obtain it.
3. *Unintended effects at the level of the field*: At this level, the HCB had identified two types of possible risks:
  - Unintended effects associated with *combining targeted modifications (multiplex genome editing) to derive new traits*: The HCB recommended mitigation of these potential risks by monitoring the novel trait in the field with regard to ecological, agro-ecological, economic and societal impact.
  - Unintended effects *associated with potential acceleration of breeding* owing to efficiency and technical ease of use of genome editing: The HCB recognised that the potential acceleration of breeding could have both positive and negative effects on the ecosystem’s functioning and dynamics. The Council thus recommended a local management to control the pace of potential agro-system change upon the roll-out (if necessary, gradual and over time and space) of the plants with novel traits; if needed, a proportional monitoring with regard to ecological, agro-ecological, economic and societal impact should be conducted.

By way of conclusion of the HCB’s analysis, Troadec summarised that potential risks of genome editing should be evaluated relative to the adoption rate of genome-edited plants. In case traceability was necessary, the introduction of a “traceability document” for genome-edited novel plants was recommended, because—unlike conventional GE/GM techniques—SDN1 and SDN2 genome editing techniques would be difficult to detect. The traceability documents could provide protocol of precautions recommended by the HCB for possible unintended effects discussed above.

## Panel discussion session 2: considerations of risk and safety

The panel discussion was moderated by the session moderator, Huw Jones, and conducted between the speakers of the session, who were joined by two additional panellists: Odd-Gunnar Wikmark from the Centre for Biosafety, Norway, and Alan Raybould from the Syngenta Crop Protection AG. At the beginning of the discussion, the two additional panellists were given the opportunity to make a statement about their main opinions on the topic of risk and safety assessment of genome-edited organisms:

Odd-Gunnar Wikmark pointed out that genome editing was a novel technology, for which novel tools for biosafety assessment should consequently be developed. He stressed that there was a lack of systemised data, upon which decisions could be based. He therefore demanded an increased effort in biosafety research and in the monitoring of genome-edited species, suggesting that this could be conducted as part of public health monitoring approaches. Such monitoring would be unspecific, but it would allow further investigation, in case an indication of a cause-effect-causality was discovered; for this to work, however, labelling and regulation were necessary.

Alan Raybould alerted the conference delegation that one essential role that policy played in the research and development of innovations was to provide the societal and economic context, in which new products were to be created and used, and to thus set the questions that needed to be asked, and the data that needed to be collected (Raybould 2019).

Policy is the beginning, not the end of the process.

(“OECD Conference on Genome Editing: Applications in Agriculture—Implications for Health, Environment and Regulation”, 28–29 June 2018)

He emphasised that the investigation of unintended effects undermined this fundamental policy role, because it turned the paradigm of hypothesis-driven research on its head and enforced *ad*-*hoc* policymaking in response to statistically significant differences that were potentially spurious.

The panel discussed the possibility to increasingly use multi-tier approaches in risk and safety assessments, in order to provide risk assessors that were obligated to enforce regulations with tools that allowed them to do so, without having to justify their decision making through requesting increasing amounts of (often unnecessary) data. It was argued that multi-tier approaches could be hypothesis driven. Not all panel members, however, supported the assumption that more data was not useful; it was very useful, they argued, but it needed to be systematised.

The panel revisited Jeffrey Wolt’s reference to the difference between “nice to know” data and “need to know” data. While the panel largely agreed that the terminology was unfortunate, it was concluded that a long history of safe commercialisation of GEOs/GMOs had shown that unintended effects did not pose large concerns, but that (suspected) unintended effects that could not be observed in reality over a long time posed large problems to risk assessors.

Challenged by the moderator to provide soundbites, the panel members concluded the following:

- A stronger leadership from policy makers and regulators was required, in order to clarify the question “what do we want this technology to achieve?”, so that the relevant science could follow from that; if this was not done, policy would be set by random, statistically significant differences in huge profiling studies.
- The ruling of the European Court of Justice on the 25^th^ July 2018 was expected to bring clarity; nevertheless, the community had to be prepared to deal with some paradoxes, under which two seemingly identical products were regulated differently.
- Most risks posed by genome editing appeared to be smaller than those posed by conventional breeding techniques, but the highly accelerated breeding enabled by genome editing still represented a significant novelty that could be addressed only with monitoring approaches at a higher ecological level, in order to identify potential risks stemming from this.
- More work in the scientific community was needed, in order to implement best practices.
- More research was needed, in order to improve the design, specificity and the product validation of genome editing techniques.
- Downstream concerns should be product focussed.

As a risk assessor, I cannot envision an appropriate risk assessment that is based simply on this technological process called ‘genome editing’.

(Panel discussion, “OECD Conference on Genome Editing: Applications in Agriculture—Implications for Health, Environment and Regulation”, 28–29. June 2018)

## Session 3: Regulatory aspects

This session looked at the regulatory questions associated with genome editing applications in agriculture, with a view to discussing approaches to address them. A series of presentations highlighted policy frameworks in specific countries: these addressed the regulatory approaches to genome editing, including legal definitions of genetic engineering/modification in relation to genome editing and risk assessment considerations, taking into account the safety of plant breeding practices and existing regulations of agricultural products.

The session was moderated by François Pythoud, Ambassador and Representative of Switzerland to FAO, IFAD and WFP, who said that the discussion at this OECD conference on genome editing was strongly linked to the FAO’s discussion in relation to the “Agenda 2030″ on global agricultural and food policy, which considered sustainable agriculture and food systems central to sustainable development. “We need a transformational change in the way we are managing food and agricultural systems, and for this, the role of innovation is critical,” he pointed out.

### Argentina

Martin Lema from the Biotechnology Directorate at the Ministry of Agro-Industry in Argentina provided an overview of the relation between Argentina and genetically engineered/genetically modified (GE/GM) crops (Lema 2019). He commenced by outlining the long history his country had with the cultivation of GE/GM crops, and the solid regulatory system this history was based on: as one of the “six founder countries”, Argentina had commercialised GE/GM crops since 1996, representing the third largest grower of GE/GM crops, with 23 million hectares. Argentina was the world’s first ranking exporter of soya-oil and -meal, the second of corn grain and the third of soy grain, and continuously reported positive economic and production impacts, as well as positive effects on agricultural sustainability.

Argentina’s regulatory framework for GMOs was based on the country’s membership of a number of international organisations and agreements (incl. CODEX, WTO SPS/IPPC), combined with its current effort to ratify the Cartagena Protocol. The National Commission of Agricultural Biotechnology (CONABIA) is a body for evaluation and consultation of GMOs that has been assigned by FAO to a Centre of Reference for biosafety of GMOs.

In 2015, Argentina had become one of the first countries to pass a resolution on new breeding techniques (NBTs); it exempted some genome-edited products as GMOs based on the criteria that were consistent with the definition of a living modified organism in the Cartagena Protocol:

[…] organism that possesses a novel combination of genetic material obtained through the use of modern biotechnology; […] “Modern biotechnology” means […] In vitro nucleic acid techniques, including recombinant deoxyribonucleic acid (DNA) and direct injection of nucleic acid into cells or organelles, […]

(Cartagena Protocol on Biosafety)

The regulatory classification criteria whether or not an NBT was a GMO in Argentina were primarily the involvement of rDNA:

1. The indicated use of rDNA leads to the assumption of a GMO, which requested the developer to ask for the determination of regulatory status.
2. A line-by-line process of candidates for NBT approval processes, for example, base information on the overall breeding process, genetic changes, trait, and bred-out of helper transgenes (or ‘effectors’) was required.

Officials furthermore offered developers the possibility of a putative assessment (an early consultation procedure) at the design stage, so necessary molecular and phenotypical characterisations could be anticipated for a hypothetical product.

Lema presented a set of preliminary statistics on the regulatory decisions his ministry had taken on NBTs in the past 3 years. Since the launch of the NBT resolution, 12 cases had been assessed, the majority of which had been at the design stage. He pointed out that the origin of applicants differed remarkably from that of the applications concerned with “traditional” GMOs. While the latter field had been dominated by a large number of applications from multinationals, followed by some applications from foreign SMEs, and close to no applications by national public research institutes and local SMEs, the order was almost reversed for NBTs: half of all cases had been brought forward by national public research institutions and SMEs, followed by a significant number from foreign SMEs and a small number from multinationals. Based on these statistics, Lema predicted that his ministry would probably observe a drop in applications for traditional GMOs and a steep increase in those involving NBTs, and that a broader range of NBT products would be available including animals and micro-organisms.

Lema pointed out that the country’s assessment procedure was specifically concerned with avoiding any regulatory gap that might arise from applications for market approval in Argentina for products that had been regulated differently or not at all in other countries. He specifically noted that his ministry had found that the question of detectability of NBT products did not require special considerations, since they had noted NBTs were “as easy (or as difficult) to detect as GMOs”.

By way of a conclusion, Lema showed a map of the world, indicating similarities that Argentina’s regulatory approach to genome editing exhibited vis-à-vis selected countries: these similarities ranged from the sharing of the same overall criteria (in the case of Brazil, Colombia and Chile) to coincidental decisions (in the case of Canada and USA).

### Australia

#### The Australian gene technology scheme

Peter Thygesen from the Office of the Gene Technology Regulator in Australia introduced the overarching framework for GMO regulation in Australia (Thygesen 2019), consisting of: (a) the *Gene Technology Act 2000 (GT Act)*, and (b) the *Gene Technology Regulations 2001 (GT Regulations)*, as well as an inter-governmental Gene Technology Agreement between the Federal, State and Territory governments of Australia (*GT Scheme*). The main objectives of the latter were that:

- regulations should be based on a scientific assessment of risks undertaken by an independent regulator;
- regulatory burden should be commensurate with the risk;
- regulations should be able to be amended to respond to the development of gene technologies; and
- regulation should provide an efficient and effective system for the application of gene technologies.

The Australian GMO regulatory scheme was integrated with other regulatory schemes, including for human food (Food Standards Australia New Zealand), which would be covered in the second half of the Australian presentation by Lisa Kelly.

Thygesen explained that Australia used a process trigger to determine whether an organism was a GMO or not under the GT Act, in which the definitions of gene technology and a GMO were broad and inclusive. A GMO was defined as an organism modified by gene technology, where “gene technology” was essentially “any technique for the modification of genes, or other genetic material”. The GT Regulations excluded the breeding methodologies, for example, radiation, chemical mutagenesis, somatic cell nuclear transfer and protoplast fusion.

He went on to describe one of the main points of uncertainty in the Australian GMO regulation: whether under one of the exclusions from the definition of GMO, “a mutant, in which the mutational event did not involve the introduction of any foreign nucleic acid (that is, non-homologous DNA, usually from another species) (Schedule 1, Item 1)”, organisms developed with some genome editing techniques would be classified as GMOs or not.

The Australian approach to solve the regulatory ambiguity was underway:

1. In 2016, the Gene Technology Regulator initiated a Technical Review of the GT Regulations (GT Regulations Review).^13^
2. In 2017, a review of the GT Act and the GT Scheme (GT Scheme Review) had commenced; while this review was still underway.^14^

The GT Regulations Review proposed the following amendments, designed to stay within the existing definitional process trigger of the GT Act; the proposed distinction focussed on the question, whether or not template-guided repair had been used in the GT process:

- *not regulated (i.e. not GMOs)*: SDN1, null segregants;
- *not regulated (i.e. not gene technology)*: radiation & chemical mutagenesis, somatic cell nuclear transfer, protoplast fusion, exogenous application of RNAi molecules; and
- *regulated (i.e. GMOs)*: SDN2, ODM (oligonucleotide-directed mutagenesis), SDN3.

#### Regulation of GM food in Australia

Lisa Kelly from Food Standards Australia New Zealand (FSANZ) explained that in Australia, GM food was regulated under the food regulatory system; the regulatory arrangements were shared between Australia and New Zealand, while organisms fell under the relevant separate regulations of both countries (cf. Peter Thygesen, above) (Kelly 2019). She pointed out that FSANZ did not develop policy, and played no role in the enforcement of regulations; it was a statutory agency that developed food standards under the Australia New Zealand Food Standards Code.

In 1999, both Australia and New Zealand had adopted the Standard 1.5.2. on “Food produced using gene technology”, which established: pre-market safety assessment and approval system for food produced using gene technology, and mandatory labelling requirements for approved food produced using gene technology. It predated the establishment of Australia’s Gene Technology Scheme (cf. Peter Thygesen, above), and therefore, used a different definition of gene technology. The definition in the Code had been based on the use of recombinant DNA techniques, thus representing a process trigger, which hitherto distinguished between “conventional breeding” techniques (for example, cross-breeding and selection, mutation breeding of plants, cell culture techniques), which had a presumption of safety based on their history of safe use and did thus not require pre-market approval, and all gene technologies (i.e. recombinant DNA techniques, transgenesis), which required a formal risk assessment and pre-market approval.

Based on the ambiguity if recent SDN1 and SDN2 types of edits and null segregants fell within the scope of the standard, FSANZ had initiated a review in June 2017, aiming to clarify the following questions^15^:

- whether the current definitions remained fit for purpose given the emergence of other techniques (incl. genome editing); and
- whether there was justification in terms of risk for subjecting derived foods to pre-market safety assessment and approval.

With a view to genome editing in particular, risk considerations of the review focussed on the following questions:

- What sort of food products were likely to be developed?
- How did these compare to conventional foods already in the food supply with a history of safe use?
- What was the potential to develop products with novel food risks?
- Could a presumption of safety to food derived using genome editing be applied?

The review was still underway, but a preliminary report had been published in the meantime.^16^

### Canada

Christine Tibelius from the Plant Health Science Division of the Canadian Food Inspection Agency presented an overview of the “Canadian Regulatory Aspects of Gene Editing Technologies” (Ellens et al. 2019). She highlighted that genome editing was an area of great interest in Canada, both in terms of trade and regulations; Canada had already hosted two international workshops on genome editing: (a) a general workshop entitled “Gene editing in an international context: scientific, economic and social issues across sectors” (co-organised by the OECD Directorate for Science, Technology and Innovation) in September 2016 (OECD 2018), and (b) an industry-led workshop on new plant breeding techniques in May 2017.

Tibelius noted that the biotechnology-related regulatory oversight in Canada was rather complex (no fewer than eight acts and policies applied to the different aspects of biotechnology products on the Canadian market), and that Canada had used existing acts and updated its regulations and policies for biotechnology starting in the 1980s and 1990s. The Canadian regulatory approach has been product-based, relying on the premise that “since there are many paths to the same result, the consistent and risk-based regulatory approach is to regulate products, not processes.”

In this light, the Canadian regulatory approach to biotechnology products had been as follows:

- *Pre*-*market safety assessment* for agriculture biotechnology products, including products produced through genome editing, only if they were novel (i.e. express a new characteristic) and could therefore pose a *new risk*.
- *No pre*-*market safety assessment* for gene-edited plant products that did not express a novel trait (“novel” meant “novel to the Canadian environment, or the food or feed supply in Canada”).

The Canadian regulatory approach was risk-based; it was characterised by a high degree of flexibility in its information requirements, which were: (a) not prescriptive, (b) subject to case-by-case considerations, and (c) outcome-based. Tibelius explained that environmental safety of novel plants was assessed with regard to (a) potential for weediness, (b) consequences of gene flow, (c) potential to become a plant pest, (d) impact on non-target organisms, and (e) impact on biodiversity. If, for example, canola with herbicide tolerance were to be commercialised, the authorisation process in Canada required the applicant to submit three separate dossiers to three assessment panels: (i.e. (a) for human food use, (b) for livestock feed use, and (c) for unconfined environmental release). Approval by all three authorities had to be co-ordinated, in order to minimize the potential for unapproved products to enter the environment, food or feed supplies in Canada.

Genetically modified animals were regulated under the Canadian Environmental Protection Act for potential environmental or indirect human health concerns; there was no specific genome editing policy yet, but several inquiries had been received. The government would examine the resulting organisms for genetic changes based on a case-by-case approach.

Tibelius summarised that Canada’s product-based regulation was sufficiently flexible to include evolving technologies. Nevertheless, Canada had already identified some policy challenges raised by genome editing, and was following established consultation and feedback procedures, in order to solve potential problems of regulatory asymmetry.

During a question and answer session, Tibelius explained that a new policy regarding familiar products was being developed in Canada, which would recognise the use of formerly submitted data in order to cut down on the data required for a new submission for a similar product. “We have 20 years of experience with certain types of transgenic products; there will be a time, when these are just accepted as being part of the Canadian environment,” she added.

### European Union

Chantal Bruetschy, Head of the Biotechnology Unit, Directorate General for Health and Food Safety of the European Commission, provided an overview of the regulatory aspects concerning genome editing in Europe (Bruetschy 2019). She introduced the key elements of the EU GMO legislation. The definition of GMO covered any organism (with the exception of human beings) in which the genetic material had been altered in a way that does not occur naturally by mating or natural recombination. Exemption from the GMO legislation included mutagenesis, under the condition that recombinant nucleic acid molecules had not been used (EC 2001).

The question of whether genome editing was covered by the EU GMO legislation had recently become part of a case for the Court of Justice of the European Union (CJEU), which had the ultimate power to interpret EU legislation. The case was due to a request for clarification by the French *Conseil d’État*, which had previously received a complaint at national level. The CJEU was expected to clarify the scope of the legislation by defining the exemption (notably that of mutagenesis), to assess the validity of the legislation in relation to recent technological developments, as well as the margin of appreciation of EU Member States for exempted organisms.

Bruetschy presented the initiatives carried out by the European Commission on the topic over the last years: In September 2017, the European Commission had organised a conference on “Modern Biotechnologies in Agriculture—Paving the Way for Responsible Innovation”; the conference, which had been set up to be highly inclusive, had resulted in the following key messages:

- the high level of safety in the EU, which was one of the highest in the world, should not be compromised;
- consumer confidence was key;
- Member States played an important role in the approval process and in reaching their constituencies and communicating to their domestic markets and consumers;
- innovation was important, but it was not sufficient on its own: consumers’ and civil society’s concerns needed to be addressed;
- risk–benefit analyses should be transparent;
- all stakeholders had a responsibility (i.e. industry, academics, policy-makers and NGOs had to communicate better, delivering clear, factual information, whilst recognising that the topic should be addressed in all its complexity);
- different types of farming did and should coexist; and
- legal certainty was urgently needed.

Bruetschy then informed the audience about a number of other (scientific) expert opinions that the European Commission had recently published on the topic of biotechnology. The Scientific Advice Mechanism, an expert group of the European Commission that provided independent scientific advice to the College of European Commissioners, had issued a paper on New Breeding Techniques (NBT) in April 2017. The paper (SAM 2017) provides an analysis of NBT compared to conventional breeding and established techniques of genetic modification.

She furthermore mentioned additional scientific work at the European Commission, including the Scientific Committees opinions on synthetic biology, as well as two mandates to the European Food Safety Authority (EFSA) on synthetic biology and gene drives (SCENIHR, SCCS, SCHER 2014; SCENIHR, SCCS, SCHER 2015a; SCENIHR, SCCS, SCHER 2015b; EU 2017).

In a broader perspective, she referred to a foresight study published in 2016, which found that “technology uptake” was one of nine important drivers in food systems, and that this driver could interact positively or negatively with other drivers, such as societal value (Mylona et al. 2016).

Innovation can be successful, if above all it is safe and then also if it has societal advantages, can bring benefits for the future, and can be clearly communicated.

(Chantal Bruetschy, “OECD Conference on Genome Editing: Applications in Agriculture—Implications for Health, Environment and Regulation”, 28–29 June 2018)

Regarding the expected future of genome editing in the EU, Bruetschy highlighted that consumers’ trust was of paramount importance for its success in agriculture.

During the brief Q&A session following her presentation, Bruetschy noted that the EU regularly authorised GMOs, which was criticised by certain stakeholders and that dialogues with all stakeholders, including at national level, will be necessary to get a common understanding of all the issues at stake.

### India

Murali Krishna Chimata from the Ministry of Environment, Forest and Climate Change, India provided the conference with an overview of the existing biosafety regulatory framework, the status of GE/GM crops and the possible regulation of new gene technologies in India (Chimata 2019), highlighting that India had been one of the first countries to pass a biosafety regulation. India’s 1989 “Rules for the manufacture, use, import, export and storage of hazardous microorganisms/genetically engineered organisms or cells” covered the entire spectrum of activities relating to research, development and use of GMOs and their products including new gene technologies (such as genome editing).

The 1989 Rules defined “gene technology” as “the application of genetic engineering including self-cloning and deletion as well as cell hybridisation, where ‘genetic engineering’ means the technique, by which heritable material, which did not usually occur or would not occur naturally in the organism or cell concerned, generated outside the organism or the cell is inserted into said cell or organism. It shall also mean the formation of new combinations of genetic material by incorporation of a cell into a host cell, where they occurred naturally (self cloning), as well as modification of an organism or in a cell by deletion and removal of parts of the heritable material.”

The rule was being implemented by three different agencies, divided into six statutory committees; the approval and implementation process was supported by a number of guidelines on: (a) contained use, (b) confined field traits, (c) food safety assessment, and (d) environmental safety assessment. Over 85 crop species were currently under various stages of R&D in India (Warrier and Pande 2016), and three had been approved or were awaiting approval: (a) BT-cotton was the only crop that had been fully approved, (b) BT-brinjal (egg plant) had been subjected to a moratorium, due to an adverse public reaction, in the middle of its approval process, and (c) transgenic mustard was awaiting approval.

Chimata pointed out the importance of activities for public awareness and information dissemination: many publications aimed to promote understanding about biotechnology and biosafety, and training programmes; these were organised for different stakeholders such as custom officers, agricultural officers, plant quarantine and even media and farmers. For better understanding and wider dissemination, publications were translated into regional languages.

India had rules that new gene technologies (including genome editing) would be regulated within the existing regulatory framework. Nevertheless, the subject was still under investigation by the relevant regulatory agencies, and appropriate guidelines and Standard Operating Procedures (SOPs) were being drafted and would be published soon.

During the Q&A session following his presentation, Chimata pointed out that the moratorium on the approval of BT-brinjal was a political issue; India’s neighbour Bangladesh, meanwhile, had approved the growing and commercialisation of the plant.

### United States

Sally McCammon from the Animal and Plant Health Inspection Service at the US Department of Agriculture, Kathleen Jones from the Center for Veterinary Medicine at the US Food and Drug Administration, and Mike Mendelsohn from the Biopesticides and Pollution Prevention Division at the US Environmental Protection Agency jointly presented the “Innovation and U.S. Regulation of the Products of Agricultural Biotechnology” (McCammon and Mendelsohn 2019).

#### US regulation of biotechnology products

McCammon pointed out that the US regulated all biotechnology products under existing laws that provided a basic network of agency jurisdiction. In 2015, however, a modernisation of the regulatory system for biotechnology products had been initiated, whose main goal was to “[e]nsure public confidence in the regulatory system and improve transparency, predictability, coordination, and efficiency of the regulatory system”. This review had resulted in two key documents:

1. 2017: Update to the Coordinated Framework (OSTP 2017); and
2. 2016: National Strategy for Modernizing the Regulatory System for Biotechnology Products (OSTP 2016).

The responsibility for the implementation of the regulation was spread over three separate agencies:

- USDA Animal and Plant Health Inspection Services (APHIS):
  - protection of plant health; and
  - safety of veterinary biologics.
- US Environmental Protection Agency (EPA):
  - regulation of plant-incorporated protectants (PIPs) and bio-pesticides;
  - safe use of new pesticides; and
  - safe use of chemicals.
- US Food and Drug Administration (FDA):
  - safety of food, food additives and feed; and
  - safety of veterinary and human drugs, and human biologics.

In addition to the national strategy and update to the coordinated framework, a report was commissioned and published in 2017; Preparing for Future Products of Biotechnology (NASEM 2017).

The most recent call to action, however, had come from the current US Administration, and had the purpose “[t]o identify legislative, regulatory, and policy changes to promote agriculture, economic development, job growth, infrastructure improvements, technological innovation, energy security, and quality of life in rural America.”^17^ The report issued by the subsequently formed “Task Force on Agriculture and Rural Prosperity” of the USDA listed five indicators of rural prosperity (USDA 2017). Among these, the indicator of “Harnessing Technological Innovation” stipulated the development of a “stream-lined, science-based regulatory policy for biotechnology”, aiming to:

- coordinate federal regulation of biotech products;
- coordinate interagency action through the Office of Science and Technology Policy (to be accessible for small and mid-sized innovators and to protect consumers); and
- expedite commercialization of biotech products.

#### USDA APHIS

McCammon explained that at APHIS, oversight authority was granted under the Plant Protection Act of 2000 (PPA). Under the PPA were regulations of the movement (i.e. permits for, or notification of, import, interstate movement, and environmental release) of regulated articles (living organisms that had been genetically engineered and involved a plant pest as either a donor, recipient or a vector). In 2017, the cumulative number of APHIS-authorised permits or notification had exceeded 350 concerning organisms developed using site-directed nucleases (incl. TALEN, ZFN, CRISPR) and *Agrobacterium* vectors in many cases.

Under the “Am I regulated” (AIR) process, APHIS encouraged developers to submit letters of enquiry, if they were not sure that their product fell under the relevant regulation.^18^ All letters were considered on a case-by-case basis and answered accordingly.

In March 2018, the US Secretary of Agriculture issued a statement to clarify “USDA’s oversight of plants produced through innovative new breeding techniques, including techniques called ‘genome editing’.” The statement did not change the existing USDA-APHIS biotech regulation (7 CFR Part 340)”, and stipulated that “Many genome-edited plants do not meet the regulation criteria to be subject to this regulation.”

In practice, this meant that “[o]rganisms with the following alterations would not be considered regulated under the USDA proposed approach: deletions, single-base-pair substitutions, introduction of sequences from sexually compatible plant relatives and complete null segregants.”

McCammon emphasised that the clarification statement had been specifically issued, in order to: (a) help promote international regulatory compatibility (to minimise trade disruption), and to (b) engage with stakeholders.

#### US FDA

Kathleen Jones from the Center for Veterinary Medicine at the US Food and Drug Administration, presented the “FDA activities related to Agricultural Biotechnology Products”. She noted that the regulatory status of a food (and feed) in the US was “dependent upon the objective characteristics of that food, independent of the methods used to develop the food”. The basic underlying policy had been outlined in a 1992 Statement:

“Section 409—Food Additives:

- New components of food will be regulated as additives if they are not generally recognized as safe (GRAS), subject to certain exceptions.
- Food additives require premarket review and approval before they can be lawfully marketed. The safety standard for use of a food additive is reasonable certainty of no harm under the conditions of intended use in food.
- In order for use of a substance to be GRAS, there must be reasonable certainty of no harm under the conditions of intended use and general recognition of that fact.”

In January 2017, the FDA had launched a request for comments seeking public input to help inform its regulatory approach to genome-edited plant-derived foods. The commenting period ended in June 2017 and FDA was now working on a clarification of its approach.

Jones explained that the regulation of genetically engineered animals in the US was subject to:

- Federal Food, Drug, and Cosmetic Act (FD&C Act), new animal drug provisions;
- National Environmental Policy Act (NEPA); and
- 2009 FDA Guidance for Industry (GFI) #187.

Similar to its plant-derived food, the FDA’s regulation of genome-edited animals was also based on products, not on processes. According to NEPA, agencies evaluated the environmental impacts of their approvals for genome-edited animals; GFI #187 had announced that FDA evaluated the intentional genomic alteration in animals, such as the rDNA construct in a GE animal, for safety to the animal and to food and for effectiveness under its new animal drug authorities. Concerning the application of genome editing to animals, too, FDA asked the public for input in 2017 and would clarify its approach according to that input.

Jones confirmed that FDA would continue to engage with domestic and international partners.

#### US EPA

Mike Mendelsohn from the Biopesticides and Pollution Prevention Division at the Environmental Protection Agency, briefly covered the US EPA’s regulatory role, regulating plants carrying pesticide traits (plant-incorporated protectants, (PIPs)), with regard to genome-edited products:

In September 2016, EPA had indicated in the National Strategy for Modernizing the Regulatory System for Biotechnology Products that it intended to clarify its approach to pesticidal products derived from genome editing.

In 2001, EPA had exempted PIPs from sexually compatible plants that occurred naturally in the plant or that were moved through conventional plant breeding (40 CFR 174.25) from the United States Federal Insecticide, Fungicide, and Rodenticide (FIFRA) requirements (for example, for product registration/licensing and field testing). EPA had also exempted residues of PIPs from sexually compatible plants that occurred naturally in the plant or that were moved through conventional plant breeding (40 CFR 174.508) from the United States Federal Food, Drug and Cosmetic Act (FFDCA) tolerance requirements (for example, for pesticide residues in food or feed, provided the residues were not present in food at levels that are injurious or deleterious to human health).

Mendelsohn pointed out that it had been argued that PIPs identical to these exempted PIPs created using CRISPR/Cas9 and similar techniques should also fall within the 40 CFR 174.25 exemption. However, when the exemption had been written in 2001, CRISPR/Cas9 and other SDNs did not exist as genome editing techniques, so that the matter required further consideration.

He concluded by noting that the US EPA was evaluating the extent to which the current exemptions covered genome-edited PIPs and considering approaches to clarify the regulatory status of these materials.

## Panel discussion session 3: regulatory aspects

The panel discussion included the moderator and speakers from Session 3. It discussed the regulatory considerations for genome editing applications identified during the session, and outlined the similarities and differences in the regulatory approaches to agricultural products developed by genome editing tools, including the legal frameworks that existed in different countries/regions. It also considered the issues arising from a lack of global harmonisation in the regulation of genome-edited applications.

### Risk communication and public acceptance

Challenged to share some good practice on communications and other activities to secure the public acceptance, panellists noted that even in the most GMO-averse regions, some farmers were probably in favour of using genetic engineering or genome editing techniques, but they knew that there was no market for products resulting from this.

[…] apart from the scientific assessment, which must confirm the safety […], the acceptance of the market is important, whether it was the economic markets (i.e. farmers of food businesses) or the consumer market.

(Panel discussion, “OECD Conference on Genome Editing: Applications in Agriculture—Implications for Health, Environment and Regulation”, 28–29 June 2018)

It was pointed out that risk communication needed to be based on science, but that natural scientists were not well skilled to hold these communications; they tended to jump to the polarised debate, because they believed in the “deficit model”, which assumed that laypeople just needed to be given enough information to come around to understanding and supporting the technology.

There is an opportunity for empirical scientists to engage with social scientists, so that [the former] understand the communication dynamics, and so that [the resulting communication] isn’t just about the technical knowledge.

(Panel discussion, “OECD Conference on Genome Editing: Applications in Agriculture—Implications for Health, Environment and Regulation”, 28–29 June 2018)

### Regulatory information requirements

The discussion briefly returned to the question of information requirements that had been central to the previous panel session on “Risk Assessment”:

[In] the last 25 years, we have been collecting more and more and more data and demonstrably not improved public confidence.

(Panel discussion, “OECD Conference on Genome Editing: Applications in Agriculture—Implications for Health, Environment and Regulation”, 28–29 June 2018)

The panel was challenged to consider, if there was an indication that risk assessors and regulators could look at the real information requirements to make safety determinations, instead of escalating data requirements, in order to insure a public that was not at all worried about the science. The panel responded by pointing out that good risk-tiering approaches were trying to achieve exactly what had been asked: rather than following a one-sided, heavy regulatory risk assessment approach, risk-tiering approaches provided a choice in information requests. Some panellists confirmed that their jurisdictions were indeed trying to phase risk-tiering approaches into an increasing number of regulatory risk assessment processes.

It was noted that even process-triggered regulatory approaches could introduce risk-tiering, based on product/outcome-focussed risk formulations. The regulators acknowledged that risk-tiering approaches also allowed a better management of resources (at both sides of the regulator and the data provider); at a time, when regulatory systems needed to address an increasing number of applications and simultaneous deal with rapidly changing technologies, regulators needed to consider the sustainability of their own systems when conducting regulatory reviews.

### Harmonised approach to diverse regulatory systems

The delegation noted that the current patchwork of regulatory approaches to genome-edited organisms did not just pose concerns to innovation, but also to trade; genome-edited products without foreign DNA would be hard to detect and could raise regulatory compliance issue. Some representatives referred to successful outreach activities, including the sharing of information directly through publishing studies on applications, regulation and social aspects of genome editing.

We don’t need a uniform world, but we need a world that understands each other, which exchanges information, and which ultimately gives access to good food to everybody.

(Panel discussion, “OECD Conference on Genome Editing: Applications in Agriculture—Implications for Health, Environment and Regulation”, 28–29 June 2018)

Some panellists mentioned successful interchanges with its trade partners to enhance the mutual understanding on their relevant regulatory actions and biotechnology products, emphasising the importance of information exchange and a potential policy agreement at higher levels for beneficial trade policies.

The panel noted that the working groups at the OECD and the consensus documents they were creating were ideal platforms to address and mitigate such concerns:

The journey [of the OECD consensus documents] is just as important as the result.

(Panel discussion, “OECD Conference on Genome Editing: Applications in Agriculture—Implications for Health, Environment and Regulation”, 28–29 June 2018)

The moderator closed the session by pointing towards the OECD strapline: “’Better policies for better lives’—that’s what we should aim for!”

## Footnotes

1 The full conference programme can be found here: http://www.oecd.org/environment/genome-editing-agriculture/oecd-conference-on-genome-editing-programme.pdf.

2 Current clinical genome editing trials are extremely expensive one-off procedures: the development of the treatment administered in ex vivo genome editing trials cost ca. 0.5 million dollars; the trials require GMP cleanroom facilities and uses equipment worth one million dollars.

3 More information on the OECD’s Biosafety work: http://www.oecd.org/science/biotrack/.

4 OECD BioTrack Product Database: https://biotrackproductdatabase.oecd.org/.

5 There are around 200 known waxy alleles. The 30 bp deletion allele had been traced back to have first been cultivated in China for as long as 500 years; by now the allele had probably arisen independently four different times (including the latest genome editing approach utilized by Corteva).

6 More information about the Genius project: http://agritrop.cirad.fr/575824/.

7 https://www.technologyreview.com/s/600765/10-breakthrough-technologies-2016-precise-gene-editing-in-plants/.

8 Egg producers around the world had already pledged to use innovations in order to stop the culling of day-old male chicks (some as early as 2020, as was the case in the USA), but they had made it clear that any suitable method would have to work with a > 98% accuracy, allow the screening and deselection of eggs at the point off laying (prior to incubation), and be non-invasive.

9 It has been shown that exclusive breeding with polled dairy sires would increase inbreeding and decrease genetic mate: http://www.wcgalp.org/system/files/proceedings/2018/simulation-introgressionpolled-allele-holstein-breed-conventional-breeding-versus-gene-editing.pdf (accessed 28 August 2018).

10 https://www.farmanddairy.com/news/eliminating-dehorning-in-dairy-cattle/492490.html.

11 All OECD Consensus Documents in the series on “Harmonisation of Regulatory Oversight in Biotechnology” (10.1787/23114622) can be found here: https://www.oecd-ilibrary.org/environment/harmonisation-of-regulatory-oversight-in-biotechnology_23114622 (accessed: 20. August 2018).

12 All OECD Consensus Documents in the series on “Safety of Novel Foods and Feeds” can be found here: http://www.oecd.org/chemicalsafety/biotrack/consensus-documents-safety-of-novel-foods-and-feeds.htm (accessed: 20. August 2018).

13 More information on the Australian GT Regulations Review can be found here: http://www.ogtr.gov.au/internet/ogtr/publishing.nsf/content/reviewregulations-1.

14 More information on the Australian GT Scheme Review can be found here: http://www.health.gov.au/internet/main/publishing.nsf/Content/gene-technology-review.

15 More information on the FSANZ review can be found here: http://www.foodstandards.gov.au/consumer/gmfood/Pages/Review-of-new-breeding-technologies-.aspx.

16 The Preliminary Report of the FSANZ Standard review can be found here: http://www.foodstandards.gov.au/consumer/gmfood/Documents/NBT%20Preliminary%20report.pdf (accessed 20. September 2018).

17 US Presidential Executive Order on Promoting Agriculture and Rural Prosperity in America (25 April 2017): https://www.whitehouse.gov/presidential-actions/presidential-executive-order-promoting-agriculture-rural-prosperity-america/.

18 More information on the APHIS AIR process can be found here: http://www.aphis.usda.gov/biotechnology/am_i_reg.shtml.
